# Self-Efficacy for Self-Regulated Learning Across Different Stages of the COVID-19 Pandemic: A Three-Wave Study with High-School Students

**DOI:** 10.3390/bs16071242

**Published:** 2026-07-21

**Authors:** Mirella Dragone, Lucia Di Martino, Grazia De Angelis, Concetta Esposito, Dario Bacchini

**Affiliations:** 1 Faculty of Law, Giustino Fortunato University, Via Raffaele Delcogliano 12, 82100 Benevento, Italy; m.dragone@unifortunato.eu; 2 Department of Humanities, Literature, Cultural Heritage and Education Sciences, University of Foggia, Via Arpi 176, 71122 Foggia, Italy; lucia.dimartino@unifg.it; 3 Department of Humanities, University of Naples “Federico II”, Via Porta di Massa 1, 80134 Naples, Italy; concetta.esposito3@unina.it (C.E.); dario.bacchini@unina.it (D.B.); 4 Department of Psychology and Health Sciences, Pegaso University, Centro Direzionale Isola F2, 80143 Naples, Italy

**Keywords:** COVID-19 pandemic, self-efficacy for self-regulated learning, teacher support, effortful control, trajectories

## Abstract

The COVID-19 pandemic caused sudden changes in school routines, creating conditions that may have been relevant to students’ self-efficacy for self-regulated learning (SESRL). This research investigated high-school students’ SESRL during the initial lockdown (Study 1) and across three pandemic stages (Study 2). Using a cross-sectional retrospective design, Study 1 (N = 802, 32.5% males; *M_age_* [Time1 (T1)] = 16.40, *SD* = 1.52) revealed a significant difference between retrospectively reported SESRL for the pre-pandemic period and SESRL perceived during the first lockdown. Also, higher perceived teacher support and effortful control were associated with higher SESRL during the lockdown. Latent Growth Curve Analyses in Study 2 [N (T1) = 455, 31.9% males; *M_age_* (T1) = 15.37, *SD* = 1.10; 71% attrition rate] showed a declining trend for perceived SESRL over time, regardless of gender and school grade, whereas COVID-19-related life disruption showed a significant time-varying effect. Specifically, higher levels of COVID-19-related life disruption were associated with lower SESRL at each time point. These findings highlight the potential value of monitoring students’ SESRL and exploring educational initiatives that support self-regulatory skills and positive teacher–student relationships, particularly during periods of widespread educational disruption. However, the longitudinal findings should be interpreted cautiously given the substantial attrition across waves.

## 1. Introduction

The Program for International Student Assessment (PISA) report, released in December 2023 (OECD, 2023a, 2023b), documented a substantial drop in students’ academic performance. This unprecedented decline has largely been attributed to the experience of the pandemic, which profoundly disrupted adolescents’ daily lives and educational systems worldwide. More broadly, large-scale societal disruptions represent critical contexts in which students’ learning environments, access to instructional support, and psychological functioning may undergo substantial alteration. In this regard, the COVID-19 pandemic can be understood as a salient example of prolonged contextual disruption whose repercussions in the post-pandemic era, especially in the school context, are well-documented (e.g., Zhang & Gillespie, 2023).

Although a cause-and-effect relationship between the outbreak of the pandemic and the decline in school performance cannot be established, it is unquestionable that the sudden transition from traditional face-to-face to online and remote learning brought many challenges for both teachers and students (Adedoyin & Soykan, 2023), undermining their perceived confidence to successfully perform academic tasks (Berman et al., 2022; Talsma et al., 2021).

Among individual factors influencing school performance, academic self-efficacy beliefs are considered among the strongest predictors, above and beyond cognitive and socio-cultural factors (Affuso et al., 2023; Meng & Zhang, 2023; Zysberg & Schwabsky, 2021). Academic self-efficacy is a multidimensional concept grounded in Social Cognitive Theory (Bandura, 1977); it is defined as the students’ beliefs in mastering specific academic subjects and curriculum areas and in self-regulating their learning activities in school (Bandura et al., 1996). Developmental and educational research conceptualizes academic self-efficacy as a dynamic construct shaped by the continuous interaction between individual beliefs and contextual affordances. Periods of instability—characterized by uncertainty, reduced feedback, and altered opportunities for mastery experiences, which occurred during the COVID-19 pandemic—may represent critical windows for understanding how students adapt to changing learning environments over time. In this context, the Cumulative Stress Model (Sameroff et al., 1998) provides a complementary framework to Bandura’s self-efficacy theory by explaining how individuals handle prolonged adversity, such as the pandemic crisis. According to this model, when individuals are exposed to chronic, repeated stressors from multiple life domains, their adaptive abilities may progressively deteriorate, undermining their confidence in facing daily challenges. From a social-cognitive standpoint, such cumulative stressors may erode the very sources of self-efficacy identified by Bandura (1977) by reducing opportunities for mastery experiences, limiting feedback and vicarious models, and heightening negative emotional and physiological states. Self-efficacy beliefs, in turn, act as a primary buffer in this process, affecting how individuals appraise and respond to environmental challenges.

Although academic self-efficacy is commonly conceptualized as a multidimensional construct, the present study focuses on self-efficacy for self-regulated learning (SESRL), a domain-specific component which reflects students’ confidence in their ability to manage learning activities autonomously by using self-regulated learning strategies for monitoring, regulating, planning, and controlling their cognitions, motivations, and actions to achieve personal learning goals within a specific learning environment (Hemmler & Ifenthaler, 2024). This dimension is considered particularly relevant in the context of emergency remote learning, where students are required to assume greater autonomy for planning, organizing, and monitoring their own academic work.

A limited number of studies addressed this topic, mainly analyzing the role of academic self-efficacy beliefs as predictors of academic achievement during the pandemic (Berman et al., 2022) rather than the individual and contextual factors associated with students’ confidence in their ability to self-regulate learning in the context of emergency online learning. Moreover, most studies involved undergraduate students (Aguilera-Hermida et al., 2021) and have largely focused on the first pandemic lockdown (Talsma et al., 2021). Therefore, little is known about how high-school students perceived their SESRL during different stages of the pandemic and how SESRL was associated with individual and contextual experiences related to the pandemic.

The current study aims to address this gap in the literature by analyzing high-school students’ SESRL during the COVID-19 pandemic, examining its individual and contextual correlates during the first lockdown (Study 1) and its trajectories across subsequent stages of the pandemic (Study 2). Specifically, Study 1 explored students’ perceptions of SESRL during the first lockdown, compared with their retrospective pre-pandemic perceptions, and examined the associations of temperamental characteristics, teacher support, and COVID-19-related life disruption with SESRL. Study 2 extended this investigation through a longitudinal design, examining the trajectories of SESRL across three pandemic phases and the concurrent associations between COVID-19-related life disruption and SESRL over time. Together, the two studies offer a broader understanding of how students experienced and reported their SESRL throughout different stages of the COVID-19 period.

### 1.1. Influence and Sources of Academic Self-Efficacy

In the school setting, several studies have highlighted the role of perceived academic self-efficacy in predicting academic outcomes, both cross-sectionally (e.g., Dogan, 2015; Høigaard et al., 2015; Koh et al., 2022) and across time (e.g., Affuso et al., 2023; Cattelino et al., 2019; Di Giunta et al., 2013). Notably, academic self-efficacy beliefs do not merely reflect cognitive abilities but also provide an independent contribution to school performance, even after controlling for IQ and family socioeconomic status (Affuso et al., 2017).

As a multidimensional construct, academic self-efficacy operates across different domains and contexts—a student may feel highly confident in one field but not in another —and may shape his/her beliefs depending on the specific context. According to Bandura’s (1977) Social Cognitive Theory, four primary sources contribute to the development of academic self-efficacy beliefs: mastery experiences, vicarious experiences from observing others, verbal and social persuasion, and emotional and physiological states (Bandura, 1977; Usher & Pajares, 2006). Students’ academic self-efficacy increases when they obtain significant success, receive frequent and immediate feedback regarding the tasks they perform, as well as when they interpret results as dependent on their capabilities and efforts. Academic self-efficacy is also shaped by vicarious experience of observing the actions of others, especially significant peers or adults. By comparing their own abilities with the successes or failures of relevant models, students are likely to alter their academic self-efficacy beliefs. Academic self-efficacy is also influenced by the encouragement students receive from significant others such as parents, teachers, and peers they trust. Lastly, academic self-efficacy beliefs are informed by emotional and physiological states, such as anxiety, stress, fatigue, and mood, because students interpret their physiological arousal as an indicator of personal competence.

Some studies have documented the association between academic self-efficacy and teacher support (Affuso et al., 2023; L. Huang & Wang, 2023; R. Yu & Singh, 2018), which exerts a protective role in helping students to face learning difficulties, as well as between academic self-efficacy and temperamentally based self-regulatory abilities (Hughes et al., 2008; Liew et al., 2008). Specifically, students who feel supported by teachers, both in traditional (Affuso et al., 2023; R. Yu & Singh, 2018) and online learning contexts (L. Huang & Wang, 2023), are more likely to exhibit positive academic beliefs, since supportive teachers may provide both social persuasion and vicarious experiences as sources for academic self-efficacy, by supplying constructive feedback regarding students’ progress and introducing peer examples and effective coping strategies in learning activities, respectively (L. Huang & Wang, 2023). Further, as regards the role of self-regulatory abilities on students’ academic self-efficacy, effortful control is recognized as the self-regulation component of temperament, which involves flexibility, attentiveness, and persistence skills (Rothbart et al., 2011), and has been shown to play a key role in promoting academic self-efficacy and academic achievement (Hughes et al., 2008; Liew et al., 2008), both in traditional (Sofologi et al., 2022) and online learning contexts (Biwer et al., 2021).

Although several studies (e.g., Bong, 2002; Foster et al., 2016) have reported high stability in academic self-efficacy beliefs over time, they remain responsive to contextual influences. Consistent with the notion that self-efficacy is context-specific and in line with the cumulative stress perspective (Sameroff et al., 1998) according to which when multiple stressors accumulate, a downward spiral could be triggered undermining individual’s psychological adaptation, academic self-efficacy beliefs could be responsive to contextual changes (Bergey et al., 2019) and subject to variations within individuals and over time (Bandura, 1997; Talsma et al., 2020). During development, when individuals face novel tasks or experiences in their lives, academic self-efficacy beliefs are most likely to change.

### 1.2. Academic Self-Efficacy for Self-Regulated Learning During the COVID-19 Pandemic

Due to the outbreak of the COVID-19 pandemic, the sudden and unprecedented shift from traditional face-to-face to online and remote learning posed a major challenge for the school system. Despite the spread of technology-based learning experiences in schools, the large-scale implementation of online learning as a tool to cope with the emergency represented an entirely new experience for educational contexts. Most teachers, students, and families were unprepared to function successfully in the novel learning environment (Vecchio et al., 2022), given their limited prior experience with online learning (Besser et al., 2022). “Emergency remote learning” was an unplanned solution for delivering instruction when unforeseen circumstances prevented teachers from being physically present in the classroom (Hodges et al., 2020; Morgan, 2020). In this context, students were required to adapt abruptly to a novel learning environment that demanded the use of intentional self-regulation strategies and challenged their perceived self-confidence in academic performance (Biwer et al., 2021). However, to date, there is little research on the impact of the pandemic-related changes in educational practice on students’ level of academic self-efficacy, and the results were inconsistent: while some studies have found that academic self-efficacy has been negatively impacted (e.g., Berman et al., 2022), others did not find these detrimental effects (e.g., Talsma et al., 2021). For instance, Lin (2021), in a study with university students in Taiwan, found relative stability in general academic self-efficacy before and during online activities during the pandemic, and even an increase in mastery perception among students with prior online experience. Talsma et al. (2021) did not observe deleterious effects among Australian undergraduate students, concluding that academic self-efficacy beliefs were stable traits, weakly influenced by changes in academic context. Aguilera-Hermida et al. (2021), in a study with Mexican, Peruvian, American, and Turkish undergraduate students, found a greater decline in academic self-efficacy among American students than among other groups, even though they were more confident with technology. Only a few studies, to our knowledge, have examined the impact of the pandemic on academic self-efficacy in high-school students, and even fewer have focused on the self-regulatory component of academic self-efficacy. In Sweden, Berman et al. (2022) found that 43.6% of high-school students reported a worsening in academic self-efficacy, 34.6% reported both positive and negative effects of the pandemic on academic self-efficacy, and only 6.8% reported an improvement in academic self-efficacy. More specifically, the aspects of academic self-efficacy most affected by the switch to online learning were difficulties concentrating on academic tasks, reduced contact with classmates, and the obligation to study at home. Strasser et al. (2023) found that, among Chilean students, high-functioning families during COVID-19 protected their children from a decrease in academic self-efficacy, whereas no associations were found between school-related factors and academic self-efficacy. In a longitudinal study of German students, Paizan et al. (2022) found that the shift to home learning during the pandemic led to distinct trajectories in academic self-efficacy across ethnic groups. They found stable academic self-efficacy trajectories for ethnic minority adolescents and an increasing trend for ethnic majority adolescents. This finding was attributed to limited access to electronic devices among minority ethnic groups and to greater parental support for managing online learning among the majority group, which compensated for the loss of teacher-led instruction.

The aim of the present study was to gain insight into high-school students’ adaptation to pandemic-related changes in the learning experience, specifically focusing on their SESRL, which the previous literature (e.g., Biwer et al., 2021) identified as crucial in successfully addressing the challenges posed by emergency remote learning.

### 1.3. The Italian Context at the Time of the COVID-19 Pandemic

Italy was the first European country where the COVID-19 pandemic spread quickly, leading to the early imposition of restrictive lockdown measures for the entire population. The transition from traditional face-to-face to online and remote learning started in March 2020 and continued until June 2020, corresponding to the end of the 2019–2020 school year. School activities resumed in person in September 2020, but just a month later, in October 2020, there was a new, dramatic peak of the pandemic, and teaching returned to online delivery. In spring 2021, fragmented attempts to return to in-person learning were hampered by recurrent waves of infections affecting students and teachers. For this reason, also during the 2020–2021 school year, most of the lessons were held online. In contrast, the 2021–2022 school year (beginning in September 2021) mainly involved in-person learning, which became blended learning when a teacher or a student tested positive for COVID-19. During in-person learning, safety devices (e.g., masks, distanced school desks) helped minimize contact among students and between students and teachers until June 2022.

### 1.4. Overview of Studies

The overall aim of the current study was to examine how high-school students perceived their SESRL across different stages of the COVID-19 pandemic and to investigate its associations with individual and contextual experiences related to the pandemic. Drawing on the previous literature suggesting that the sudden transition from traditional face-to-face to emergency remote learning posed several challenges for students and schools (Besser et al., 2022), we hypothesized that students would perceive a decline in their ability to cope with academic tasks. Moreover, we hypothesized that the more students perceived the pandemic experience as disruptive, the greater the perceived decline in SESRL would be. We explored these hypotheses through two complementary studies. In the first study, we examined how students reported their SESRL in the first wave of the COVID-19 pandemic compared with their retrospectively reported pre-pandemic SESRL, while also considering the concurrent roles of COVID-19-related life disruption, perceived teacher support, and effortful control. In the second study, we analyzed data from a three-wave cohort study to investigate patterns of SESRL across three different stages of the pandemic (2020–2021–2022), which differed in terms of health protection rules as well as organization of the learning experience. In both studies, particular attention was paid to the association between perceived COVID-19-related life disruption and SESRL, as well as to the potential role of gender and school grade.

## 2. Study 1

### 2.1. Aims and Hypotheses

The main aim of this study was to investigate whether students attending high schools reported different levels of SESRL at the early stage of the pandemic (Spring 2020) compared with their retrospectively reported pre-pandemic SESRL. Furthermore, we examined the association of COVID-19-related life disruption, perceived teacher support, and effortful control with SESRL during the early stage of the pandemic, while accounting for retrospectively reported pre-pandemic SESRL. Consistent with the literature mentioned above (e.g., Berman et al., 2022), we expected students to report lower SESRL during the lockdown period than when retrospectively referring to the pre-pandemic period (Hypothesis 1). Notably, the current study cannot be considered a genuine pre-post pandemic design since, unlike longitudinal prospective measurements, students were asked to rate both their current and their perceived pre-pandemic SESRL at the same assessment time (i.e., the first COVID-19 lockdown). As a result, their subjective retrospective perceptions of pre-pandemic SESRL may be vulnerable to recall bias, implicit theories of change, and present-state effects (e.g., Blome & Augustin, 2015). We also hypothesized that students who reported higher life disruption caused by the pandemic, lower perceived teacher support, and lower effortful control would report lower levels of SESRL during the lockdown period (Hypothesis 2). Given the limited literature examining students’ SESRL during the COVID-19 pandemic, moderation analyses were conducted on an exploratory basis. Specifically, we explored whether the associations between retrospectively reported pre-pandemic SESRL and SESRL during the lockdown varied according to adolescents’ levels of COVID-19-related life disruption, perceived teacher support, and effortful control.

### 2.2. Participants and Procedure

The participants were 802 Italian adolescents (261 males and 541 females; *M*_age_ = 16.40, SD = 1.52) enrolled in the first (i.e., 9th grade, 16.8%), second (i.e., 10th grade, 19.8%), third (i.e., 11th grade, 20.1%), fourth (i.e., 12th grade, 22.4%), and fifth (i.e., 13th grade, 20.8%) years of five high schools located in the metropolitan area of Naples, in Southern Italy. In the Italian educational system, these grades correspond to the five years of upper secondary education (high school), typically attended by students aged approximately 14 to 19 years. The sampling procedure did not utilize a full probability sampling design; rather, participants were recruited from a university-school collaborative network, and the classrooms involved in the research project were selected by school representatives. Data collection took place during the first pandemic lockdown (April–May 2020) through an online survey via the Qualtrics platform. At the time of the survey, Italian educational institutions were struggling with the restrictions imposed by the government to prevent the spread of the pandemic, and for all school activities, the abrupt shift from face-to-face to emergency remote learning was mandatory. A total of 1252 students accessed the survey through a web link distributed independently by the school representatives. Accordingly, information regarding the exact number of students who received the invitation or declined participation was not available, and therefore a response rate could not be calculated. For the purposes of the present study, only participants who provided valid data on at least 80% of the questionnaires relevant to the study variables were retained, resulting in a final analytic sample of 802 adolescents (64.1% of those who accessed the survey).

After an agreement was established with the school head, written informed consent was obtained from parents or legal guardians to allow adolescents to participate in the study. Adolescents gave their electronic informed consent prior to filling in the online questionnaire. All participants were informed of the voluntary nature of their participation and their right to withdraw from the research at any time, without explanation and without negative consequences for the participant.

The study’s protocol was reviewed and approved by the University Research Ethical Board (protocol code n. 21/2020) before data collection. The approved procedures included online recruitment, parental informed consent, adolescent assent, the protection of participant anonymity, and the use of self-generated identification codes to link responses across waves. The study was carried out in accordance with the recommendations of the Italian Psychological Association. The American Psychological Association’s ethical standards regarding research with human subjects were followed throughout the research design and implementation.

### 2.3. Measures

#### 2.3.1. Academic Self-Efficacy for Self-Regulated Learning as Retrospectively Reported Before the Pandemic and During the COVID-19 Pandemic Lockdown

Participants were asked to respond to three items from the Academic Perceived Self-Efficacy Scale (Pastorelli et al., 2001), indicating how capable they felt of conducting the proposed learning activities using a five-point Likert scale from 1 (‘cannot do at all’) to 5 (‘most certainly can do’). Given the growing demand for autonomy and self-regulated learning due to the unique characteristics of emergency remote learning (Biwer et al., 2021)—and in consistent with previous studies (e.g., Pribeanu et al., 2023) supporting the conceptualization of academic self-efficacy as a multidimensional model that includes, among others, self-efficacy for self-regulating learning (SESRL)—our aim was to assess such specific dimension, defined as the ability to plan and organize academic activities and keep up with academic work, thereby structuring learning environments and motivating oneself to conduct one’s schoolwork. Therefore, we selected three key items—i.e., ‘the ability to concentrate on school subjects’, ‘organize schoolwork’, and ‘remember the contents of teachers’ lessons’—that theoretically align with Bandura’s (1977) conceptualization of academic self-efficacy and specifically capture students’ SESRL. The other excluded items from the original academic self-efficacy scale by Pastorelli et al. (2001) covered different domains of academic activities, including the students’ beliefs in their capability to master different areas of coursework (e.g., ‘How well can you… learn general mathematics?’) or the self-efficacy beliefs to meet parental and teacher expectations (e.g., ‘How well can you… live up to what your parents expect of you?’), as well as scenarios that were not specifically relevant to the current emergency online learning (e.g., ‘use the library to get information for class assignments?’).

The items were administered twice: the first time asking students to answer referring to the period before the health emergency; the second time asking students to think about the current moment, corresponding to the home confinement following the outbreak of the pandemic.

The factor structure of the scale was tested through Confirmatory Factor Analysis (CFA; see Table S1 for further details). The results supported a two-factor solution (i.e., SESRL as retrospectively reported before the pandemic and SESRL during the pandemic lockdown), χ^2^ (5) = 10.78, *p* = 0.06; CFI = 0.99, RMSEA = 0.04, 90% C.I.s [0.00, 0.07], SRMR = 0.02.

Two global scores of SESRL, as retrospectively reported before the pandemic and during the first pandemic lockdown, were obtained by averaging item ratings.

Cronbach’s α coefficients were 0.79 and 0.87, respectively, indicating a good level of internal consistency.

#### 2.3.2. COVID-19-Related Life Disruption

The impact of the pandemic on adolescents’ lives has been measured using a questionnaire developed ad hoc for the current study. Participants were asked to rate the extent to which the outbreak of the pandemic had disrupted their routines in nine life domains—i.e., ‘Please rate how much the COVID-19 outbreak has been disruptive to you personally. Think about your…relationships with parents and with peers, romantic relationships, school activities, free time, physical and psychological health, academic performance, and family economic status—using a four-point Likert scale from 1 (‘not at all’) to 4 (‘a lot’). The conceptualization of the measure as capturing COVID-19-related disruption in multiple domains of adolescents’ daily life was based on ecological and cumulative stress perspective (Sameroff et al., 1998) as well as on previous studies (e.g., Louie et al., 2023) carried out during the pandemic, which suggested that experiencing chronic stressors from different areas of life can combine to influence individuals’ overall perceptions of disruption and worsen their ability to adapt them. Specifically, our aim was to operationalize the impact of multiple daily-life COVID-19 stressors, as perceived by adolescents. This was achieved by including in the scale all the crucial ecological domains that could have tapped their perceptions of pandemic impact as a stressful, pervasive event, rather than limiting it to specific life domains. To examine the dimensionality of the scale, an exploratory factor analysis was conducted prior to the CFA. Although alternative solutions were explored, the one-factor solution was retained because it provided the most parsimonious and interpretable representation of the data, whereas multi-factor solutions showed substantial cross-loadings and limited interpretability. The results of the CFA confirmed the adequate goodness of fit of the single-factor model, with all items loading on one latent factor, χ^2^ (26) = 99.94, *p* < 0.001; CFI = 0.93, RMSEA = 0.06, 90% C.I.s [0.05, 0.07], SRMR = 0.04 (see Table S2 for further details). A global score was obtained by averaging item ratings, and Cronbach’s α was 0.73, indicating good reliability.

#### 2.3.3. Teacher Support

Teacher support during lockdown was measured with the teacher-related subscales from the Classroom Life Scale (Johnson et al., 1985), which allowed us to assess both academic (four items, e.g., ‘My teachers want me to do my best in schoolwork’) and personal (four items, e.g., ‘My teachers really care about me’) dimensions of support. Participants rated each item on a five-point Likert scale from 1 (‘never’) to 5 (‘always’). The factor structure of the scale was tested through CFA. Consistent with previous studies (Esposito et al., 2021), our results confirmed the adequate goodness of fit of the higher-order structure of the scale, with one second-order factor (i.e., teacher support), χ^2^ (19) = 78.66, *p* < 0.001; CFI = 0.98, RMSEA = 0.06, 90% C.I.s [0.05, 0.08], SRMR = 0.03.

For the purposes of the present study, we operationalized teacher support as a global measure by averaging all item ratings related to both academic and personal dimensions. The instrument demonstrated good reliability, with a Cronbach’s α of 0.89.

#### 2.3.4. Effortful Control

Temperamental effortful control (EC) was assessed using the Early Adolescent Temperament Questionnaire-Revised (EATQ-R; Ellis & Rothbart, 2001; Snyder et al., 2015), which is also frequently used in the Italian context (Esposito et al., 2020). Participants rated each of the 15 items on a five-point Likert scale from 1 (‘almost never true’) to 5 (‘almost always true’), and a global score was obtained by averaging item ratings related to the subdimensions of the activation control (e.g., ‘If I have a hard assignment to do, I get started right away’), attention control (e.g., ‘I pay close attention when someone tells me how to do something’), and inhibitory control (e.g., ‘When someone tells me to stop doing something, it is easy for me to stop’) scales, after recoding reverse scored items.

To examine the dimensionality of EC, a CFA at the item level was conducted. The model showed an acceptable fit, χ^2^(75) = 310.95, *p* < 0.001, RMSEA = 0.06, 90% C.I.s [0.06, 0.07], SRMR = 0.05. The CFI was not optimal (0.874) but consistent with prior research, which suggests that the factorial structure of the EATQ-R is complex and not adequately represented by simple item-level models (Lawson et al., 2021; Snyder et al., 2015).

The instrument demonstrated good reliability, with a Cronbach’s α of 0.76.

### 2.4. Data Analysis

The statistical analyses were carried out in IBM SPSS Statistics version 21 (IBM Corp.; Armonk, NY, USA). Preliminarily, we checked our data for outliers and assessed the univariate normality of the variables by examining skewness and kurtosis values. Additionally, since participants were nested within five schools, intraclass correlation coefficients (ICCs) were estimated to evaluate potential clustering effects. ICC values were low (<0.05), indicating limited between-school variability. Therefore, school-level clustering was not explicitly modeled in the subsequent analyses.

Then, we carried out a Repeated Measures Univariate Analysis of Variance (RM-ANOVA) to compare students’ levels of SESRL during the first lockdown and retrospectively reported pre-pandemic SESRL (‘within-subjects’ factor). Gender (1 = male vs. 2 = female) and school grade (9th to 13th grade) were introduced in the model as ‘between-subjects’ factors. The partial eta-squared (η^2^p) statistic was used to establish the effect size, which was interpreted as follows (Cohen, 1988): small (0.01), medium (0.06), and large (0.14). A *p*-value < 0.05 was adopted for all statistical tests. Pearson correlations were then computed to examine the associations between the study’s variables. Lastly, to test the main study’s hypothesis regarding the association between COVID-19-related life disruption, perceived teacher support, and effortful control (independent variables) and SESRL as perceived by students during the first lockdown (dependent variable), accounting for retrospectively reported pre-pandemic SESRL, a hierarchical multiple regression analysis was conducted. Prior to testing the hypothesized model, all assumptions of multiple linear regression were assessed and found to be satisfied. Outliers were assessed using Cook’s distance; all values were below the conventional threshold of 1, indicating no influential cases. Scatterplots showed that the relationships between the continuous independent variables and the dependent variable were approximately linear. Homoscedasticity of the residuals’ variance was supported, as the residual scatterplot showed even dispersion without a clear pattern, indicating that the residuals’ variance was roughly equal (Tabachnick & Fidell, 2013). Further, multicollinearity among independent variables was examined by calculating Tolerance and Variance Inflation Factors, which were below the thresholds of 0.2 and 5.0, respectively, indicating no problematic multicollinearity (O’Brien, 2007).

Consistent with the study hypotheses, variables were entered into the regression models in three successive blocks. Retrospectively reported pre-pandemic SESRL was entered in the first step (Model 1). Gender and school grade were entered in the second step (Model 2). In the third step, COVID-19-related life disruption, perceived teacher support, and effortful control were entered simultaneously to evaluate their associations with SESRL, controlling for the effects of the control variables (Model 3).

Finally, we used the PROCESS-Macro (Hayes & Rockwood, 2020) plugin to test moderation exploratory hypotheses (Models 4–8). Interaction terms were computed as the product of the focal predictor (retrospectively reported pre-pandemic SESRL) and the moderator (COVID-19-related life disruption, perceived teacher support, and effortful control). Consistent with the default procedure implemented in PROCESS, predictors were entered in their original metric, and simple slopes were analyzed at one standard deviation above and below the mean of the moderator. Unstandardized regression coefficients are reported throughout.

### 2.5. Results

#### 2.5.1. Comparison Between Students’ Perceived Self-Efficacy for Self-Regulated Learning During the First Lockdown and Their Retrospectively Reported Pre-Pandemic Self-Efficacy

Preliminarily, we checked for the distribution of variables. No variables approached skewness > |3| or kurtosis > |10|, indicating that the data followed a normal distribution (Weston & Gore, 2006). The means and standard deviations of the study variables are available online as Supplementary Materials (see Table S3).

**Figure 1:**
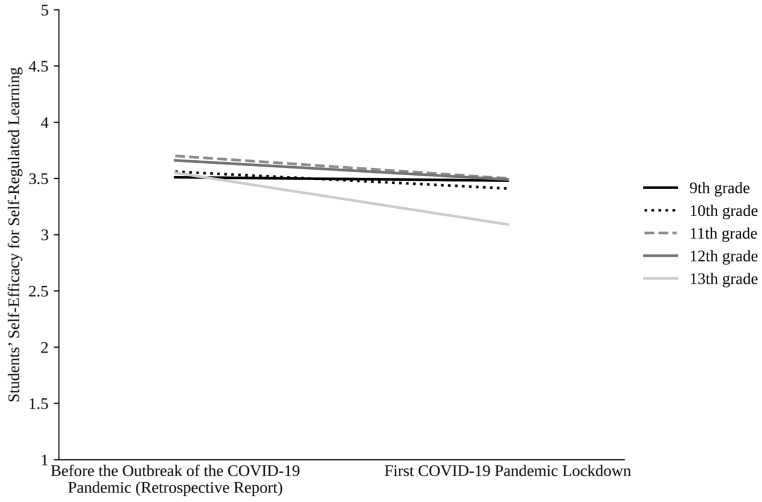
Trends for self-efficacy for self-regulated learning during the first COVID-19 pandemic lockdown and retrospectively reported pre-pandemic SESRL for all school grades.

Findings from the RM-ANOVA showed a significant Time effect (Wilks’s λ = 0.94; F(1, 792) = 47.72, *p* < 0.001, η^2^*p* = 0.06) and Time × School grade interaction effect (Wilks’s λ = 0.96; F(4, 792) = 7.29, *p* < 0.001; η^2^*p* = 0.04). As displayed in Figure 1, students reported lower levels of SESRL during the first lockdown than they retrospectively reported for the pre-pandemic period, with larger differences observed among students attending higher levels of school grade.

#### 2.5.2. Correlations Among Study Variables

Pearson correlations among all study variables are available online as Supplementary Materials (see Table S3). All the study variables were significantly associated with each other. As expected, retrospective reports of pre-pandemic SESRL and reports of SESRL during the pandemic lockdown were significantly and positively associated with each other, as well as with teacher support and effortful control. Also, both reports were negatively linked to COVID-19-related life disruption. The latter was negatively associated with teacher support and effortful control, which were positively associated with each other.

With respect to the associations with socio-demographic variables, we found that females reported significantly higher levels of retrospective pre-pandemic SESRL, and older students reported lower levels of SESRL during the pandemic lockdown.

#### 2.5.3. Regression Models: Individual and Contextual Correlates of Self-Efficacy for Regulated Learning During the First Lockdown

To test the main hypothesis of the study, a hierarchical regression analysis was performed. Given the findings from correlation analysis, we included retrospectively reported pre-pandemic SESRL, adolescents’ gender, and school grade as control variables in all models. As Table 1 shows, the main effects were included in Models 1–3, which were all significant (*p* < 0.001), explaining about 41% of the variance in SESRL during the lockdown (R^2^ = 0.41, *p* < 0.001). Specifically, the COVID-19-related life disruption had a significant negative effect (b = −0.34, *p* < 0.001, 95% C.I.s [−0.43, −0.24]), while both teacher support and students’ effortful control had positive effects (bs = 0.14 and 0.26, *p* < 0.001, 95% C.I.s [0.06, 0.22] and [0.13, 0.40], respectively).

As regards our moderation analysis, the effects of the interaction terms on the dependent variable are shown in Models 4–8. Models 6 and 7 were found to be statistically significant (*p* < 0.001 and *p* < 0.05, respectively), although the R^2^ change values indicate a very small (1%) effect size in explaining students’ levels of SESRL. More specifically, the interactions between retrospectively reported pre-pandemic SESRL and both COVID-19-related life disruption and teacher support were statistically significant (bs = −0.19 and 0.09, ps < 0.001 and 0.05, 95% C.I.s [−0.29, −0.09] and [0.01, 0.17], respectively), although they accounted for only a small proportion of the variance of SESRL over and above the direct effects.

**Figure 2:**
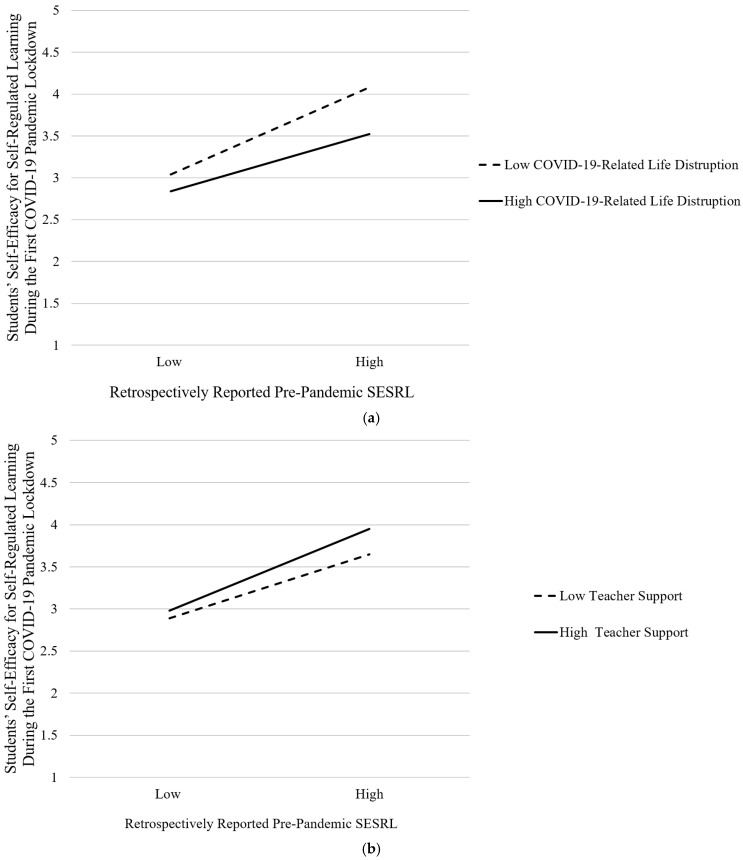
Plots of the significant interaction effects of predictors on self-efficacy for self-regulated learning (SESRL) during the first COVID-19 pandemic lockdown: (**a**) The moderating role of COVID-19-related life disruption in the association between retrospectively reported pre-pandemic SESRL and SESRL during the first lockdown; (**b**) The moderating role of teacher support in the association between retrospectively reported pre-pandemic SESRL and SESRL during the first lockdown.

Simple slopes analysis (see Figure 2a,b) showed that the association between retrospectively reported pre-pandemic SESRL and SESRL during the first lockdown varied as a function of COVID-19-related life disruption and perceived teacher support. Specifically, the association was stronger at low levels (−1 SD) of COVID-19-related life disruption and at high levels (+1 SD) of teacher support (bs = 0.62 and 0.58, *p* < 0.001, 95% C.I.s [0.53, 0.71] and [0.48, 0.67], respectively), than at high levels of negative impact of the COVID-19 pandemic and low levels of teacher support (bs = 0.40 and 0.45, *p* < 0.001, 95% C.I.s [0.31, 0.50] and [0.36, 0.54], respectively).

### 2.6. Discussion

The present study yielded two main findings. First, consistent with Hypothesis 1, students reported, on average, lower SESRL during the lockdown period compared to their retrospectively reported pre-pandemic SESRL. Second, consistent with Hypothesis 2, students’ SESRL beliefs during the COVID-19 lockdown were concurrently associated with COVID-19-related disruption in their daily lives, perceived teacher support, and temperamental effortful control. These effects were found controlling for retrospectively reported pre-pandemic SESRL, gender, and school grade.

Although a significant difference between students’ SESRL as perceived during the pandemic lockdown compared with their retrospectively reported pre-pandemic SESRL has emerged, with larger perceived differences among students attending higher school grades, it should be noted that our study cannot be considered a true pre-post design since students reported both pre-pandemic and lockdown SESRL at the same assessment. Accordingly, drawing on previous studies (e.g., Guazzini et al., 2022; Yamagata & Miura, 2023) highlighting the risk of retrospective bias during the COVID-19 pandemic, our design does not warrant causal or even reliable within-person inference; our findings should therefore be interpreted with caution. More specifically, the observed difference in students’ SESRL as perceived during the pandemic lockdown compared with their retrospectively reported pre-pandemic SESRL may reflect some biases specific to retrospective measurement (e.g., Blome & Augustin, 2015): the tendency to remember one’s own state as better or worse than it actually was and give different responses between before and after as a consequence (i.e., the “recall bias”); the tendency to reconstruct former state from a combination of one’s own current state and the personal assumptions on how one’s own state has probably changed (i.e., the “implicit theory of change”); and finally, the tendency to use information on one’s own current state to reconstruct one’s own former state (i.e., the “present state” bias), rather than a real change resulting from the pandemic crisis.

Consistent with Bandura’s (1997) conceptualization of self-efficacy as a context-specific construct, our findings suggest that SESRL beliefs do not represent stable personality traits but may vary based on contextual experiences, such as sudden stressful events like the pandemic, and sources of support, such as those provided by teachers during home confinement (L. Huang & Wang, 2023).

Within this unprecedented educational context, higher levels of perceived teacher support were associated with higher levels of SESRL during the lockdown, even after accounting for retrospectively reported pre-pandemic SESRL. In line with prior research (L. Huang & Wang, 2023), this finding suggests a beneficial role of teacher support in helping students maintain self-confidence while facing the exceptional challenges posed by the pandemic-related changes in education. For instance, supportive teachers may have fostered students’ engagement in academic activities and helped them not to become discouraged when faced with novel and challenging tasks; through empathy, authenticity, and the provision of emotional and informational support on the online platforms, teachers might have helped students adapt successfully to the new learning environment (L. Huang & Wang, 2023; Saefudin et al., 2021; Tannert & Gröschner, 2021).

Similarly, consistent with previous studies (Biwer et al., 2021; Hughes et al., 2008; Liew et al., 2008; Sofologi et al., 2022), our findings highlighted the key role of self-regulatory abilities in fostering students’ school-related confidence. Online learning environments often lacked the immediate support and feedback from teachers and peers that traditional classroom settings provide; as a result, students had to rely more on their own ability to focus, stay motivated, and manage their time and effort. Effortful control, an aspect of temperament encompassing precisely these skills of attention, effort, and persistence, may thus have fostered students’ confidence in their capability to manage learning autonomously.

Finally, higher levels of COVID-19-related life disruption were associated with lower SESRL during the lockdown. In line with Bandura’s (1997) view that self-efficacy beliefs are also informed by emotional and physiological states, such as anxiety, stress, fatigue, and mood, and given that the disruption measure captured adolescents’ perceptions of difficulties across multiple life domains (Louie et al., 2023), this finding may reflect a broader perception of psychological distress in students’ everyday lives, which may have influenced the way they weighted, interpreted, and integrated information when judging their academic capabilities. For instance, as suggested by Talsma et al. (2021), students experiencing distress associated with COVID-19-related changes may have interpreted it as a cue of vulnerability to perform poorly (Bandura, 1997), thus judging their own efficacy more negatively.

No differences emerged by gender. Moreover, the exploratory moderation analyses suggested that the association between retrospectively reported pre-pandemic SESRL and SESRL during the lockdown differed slightly according to levels of perceived teacher support and COVID-19-related life disruption. However, these interaction effects accounted for only a very small proportion of additional variance and should therefore be interpreted cautiously. Accordingly, these findings are best viewed as preliminary and hypothesis-generating rather than as evidence of robust moderation processes.

Despite the novel insights provided by the current study, we are aware of the severe limitation represented by the use of a retrospective measure for pre-pandemic SESRL as well as by the lack of a comparable cohort of students not impacted by the pandemic, which suggests the need to be more cautious in interpreting and generalizing the findings. Therefore, we cannot rule out that the distress caused by the outbreak of the pandemic could have amplified students’ subjective perception of a general worsening in several areas of the self, compared to the pre-pandemic period, leading to inaccurate conclusions about their perceived decline in SESRL after the outbreak of the pandemic. An additional limitation concerns the gender composition of the sample, which included a predominance of female participants. Although gender was statistically controlled in the analyses, the overrepresentation of female students may limit the generalizability of the findings.

## 3. Study 2

Whereas Study 1 examined students’ perceptions of SESRL during the initial lockdown relative to their retrospectively reported pre-pandemic SESRL, Study 2 aimed to investigate patterns of SESRL across three different stages of the COVID-19 pandemic (i.e., first wave: shortly after the declaration of the pandemic, during the first strict lockdown; second wave: 1 year after the outbreak of the pandemic, corresponding in Italy to a second lockdown following a period of reduced restriction; third wave: corresponding to the return to the face-to-face learning model even if following special safety rules). Specifically, the main research question was whether SESRL, as perceived by students during the early stage of the pandemic, remained relatively stable in the following stages, showed a recovery trend, or continued to decline over time.

Because self-efficacy beliefs are thought to be responsive to contextual conditions, we posited two alternative hypotheses concerning the trajectories of SESRL during the following stages of the pandemic. According to the first hypothesis (i.e., ‘habituation or settlement hypothesis’—Hypothesis 1a), we expected SESRL to remain stable during the second critical stage of the pandemic (i.e., during the second lockdown), with also the possibility of a gradual recovery over time. This expectation was based on the assumption that students would become more familiar with online learning methods over time, thus gradually overcoming the initial challenges associated with remote learning. Alternatively, in line with the Cumulative Stress Model (Sameroff et al., 1998) hypothesis (Hypothesis 1b) and informed by previous studies (e.g., Louie et al., 2023) showing that the more individuals experienced multiple, overlapping pandemic-related stressors (e.g., health threats, isolation, economic strain), the more likely their personal resources progressively weakened, resulting in worsened psychological adaptation, we expected a declining trajectory of SESRL across the different stages of the pandemic, gradually eroding students’ confidence in their learning abilities over time in a cascade process.

In addition, we analyzed the effect of gender and school grade as time-invariant covariates of the intercept and slope of SESRL, as well as of COVID-19-related life disruption as a time-varying covariate of SESRL at each measurement point. Consistent with previous meta-analyses (e.g., C. Huang, 2013) finding gender-based differences in academic self-efficacy beliefs and that such differences also varied with age, we expected a significant association of both time-invariant covariates with initial levels of SESRL (Hypothesis 2).

Drawing on social-cognitive theory and evidence linking contextual stressors to self-efficacy beliefs, we hypothesized a negative concurrent association between COVID-19-related disruption and SESRL across all measurement points (Hypothesis 3).

### 3.1. Participants and Procedure

Participants were recruited from the same pool of five schools participating in Study 1. Because the study followed the same cohort across three annual assessments, students enrolled in the last two years of high school at T1 were excluded from this study (N = 347), as they would have graduated before the completion of the three-wave follow-up. More specifically, in the first data collection, participants were 455 students attending grades 9 to 11 (145 males and 310 females; Mage [Time 1 (T1)] = 15.37, SD = 1.10). In the subsequent data collections, the sample included 260 students in grades 10 to 12 (86 males and 174 females; Mage [Time 2 (T2)] = 16.12, SD = 1.03) and 132 students in grades 11 to 13 (38 males and 94 females; Mage [Time 3 (T3)] = 17.50, SD = 0.98), respectively.

The three time points of data collection (1-year intervals) corresponded to different stages of the pandemic, marked by different levels of infection and death rates, as well as restrictions in various life domains. Because of COVID-19 restrictions, all measures were included in an online survey that students completed by connecting to a web link posted by the school representatives during Spring 2020 (T1), 2021 (T2), and 2022 (T3).

The procedure for the data collection was the same as already described for Study 1. To link participants’ responses across the three assessment waves while preserving anonymity, students were asked to generate a unique personal identification code based on a predefined combination of personal information known only to them. The same code was reported at each wave and was used to match questionnaires longitudinally. A record was considered successfully matched only when the identification code was identical across assessment occasions.

### 3.2. Measures

#### 3.2.1. Academic Self-Efficacy for Self-Regulated Learning During the Different Stages of the COVID-19 Pandemic

At each time point, participants were asked to respond to three items from the Academic Perceived Self-Efficacy Scale (Pastorelli et al., 2001) previously described for Study 1. A global score was obtained by averaging item ratings. Cronbach’s αs were 0.85 for T1, 0.72 for T2, and 0.79 for T3, thus indicating a good level of internal consistency.

#### 3.2.2. Time-Invariant Covariates

Demographic data were obtained from the participants at T1. Students reported their gender (1 = male, 2 = female) and school grade (from 1 = 9th to 3 = 11th grade).

#### 3.2.3. Time-Varying Covariate: COVID-19-Related Life Disruption During the Different Stages of the COVID-19 Pandemic

At each time point, participants were asked to rate the extent to which the pandemic had disrupted their daily routines in nine life domains by using the same scale previously described for Study 1.

A global score was obtained by averaging item ratings. Cronbach’s αs were 0.73 for T1, 0.75 for T2, and 0.80 for T3, thus indicating a good level of internal consistency.

### 3.3. Missing Data and Attrition Analysis

Attrition analysis showed that 195 adolescents from the original sample (T1, N = 455) were not assessed at T2 (42.9%), and 323 adolescents were not assessed at T3 (71%). Examining individual participation patterns across the three waves, 102 students (22.4%) provided data at all three time points, 158 (34.7%) were present at T1 and T2 but not at T3, 165 (36.3%) participated at T1 only, and 30 (6.6%) displayed an intermittent participation pattern, being present at T1 and T3 but not at T2.

The loss of participants across the three years of the study was primarily attributable to school dropout, errors in identification codes that made it impossible to match records across data collection waves, and difficulties in maintaining contact with participating schools after the peak of the pandemic. Notably, as reported by Gustavson et al. (2012), at 50% and 70% attrition rates, mean estimates might become extremely biased, requiring careful attention when interpreting the study’s findings. Little’s test (Little & Rubin, 2002) for Missing Completely at Random (MCAR) conducted using SPSS 21 (IBM Corp.; Armonk, NY) was not significant, χ^2^(10) = 15.494, *p* = 0.12, and *t*-tests revealed no significant differences between those who dropped out and those who remained in the study across key demographics (gender and age) and other study variables (all ps > 0.05). Density plots of the study variables further indicated comparable distributions across groups (see Figure S1 in the Supplementary Materials). Together, these findings provide no evidence of systematic missingness with respect to observed variables.

However, although Little’s test did not suggest departures from MCAR and no differences emerged on observed variables, Full Information Maximum Likelihood (FIML) estimation was adopted because it provides robust parameter estimates under the broader Missing at Random (MAR) framework. Nevertheless, these analyses cannot rule out the possibility that attrition was associated with unobserved characteristics. For example, students experiencing greater academic difficulties, lower school engagement, reduced access to educational resources, or greater family adversity may have been less likely to remain in the study (e.g., Affuso et al., 2025; Alejo et al., 2024).

### 3.4. Data Analysis

As in Study 1, ICC values were low (all < 0.05), indicating limited between-school variability. Accordingly, and given the small number of participating schools, school-level clustering was not explicitly modeled, and all analyses were conducted at the individual level.

The main analyses were conducted with Mplus 8 (Muthén & Muthén, 1998–2017). Missing data were handled using the Full Information Maximum Likelihood (FIML) method.

Preliminarily, longitudinal invariance of SESRL and COVID-19-related life disruption measures was tested through separate Confirmatory Factor Analyses (CFAs). Configural, metric, and scalar levels of invariance were assessed. Several indices were used to evaluate model fit: the chi-square statistic (χ^2^), the Comparative Fit Index (CFI; Bentler, 1990), and the Root Mean Square Error of Approximation (RMSEA; Browne & Cudeck, 1993). The Satorra–Bentler chi-square difference test (ΔSBχ2) was used to compare the fit of nested models (Satorra, 2000). A non-significant chi-square difference test of model fit indicates that the constraints do not result in a worse fit; thus, the more constrained, more parsimonious model was preferred in this case. Conversely, when the more constrained model was rejected, a less restrictive model of partial measurement invariance was tested in which, in accordance with modification indices, equality constraints on one or more parameters were relaxed until the change in the fit was no longer significant.

Next, concurrent and longitudinal associations between the study’s variables were examined using Pearson correlations. Subsequently, Latent Growth Curve (LGC) models (Duncan, 2011) were used to test the change in SESRL over time. Within this framework, the repeated observed variables can be used to estimate the unobserved underlying trajectory defined by two latent growth factors—the intercept and the slope. To examine the growth over time of the SESRL, a series of LGC models of increasing complexity were constructed. First, three alternative two-factor (intercept, slope) unconditional LGC models were run. In the first model, no change over time was postulated (only intercept); in the second model, a significant linear change over time was postulated (intercept and linear slope); in the third model, a significant nonlinear change over time was postulated (intercept, linear, and quadratic slope). Time was coded as 0, 1, and 2, corresponding to T1 (2020), T2 (2021), and T3 (2022), respectively. Under this parameterization, the intercept represents the estimated level of SESRL at T1, whereas the slope represents the average linear rate of change across the three assessment occasions.

The fit of these three competing models was compared using the Bayesian Information Criterion (BIC) and the Akaike Information Criterion (AIC). Overall, models with smaller values of BIC and AIC are preferred. Also, the statistical significance of the growth parameters was considered in order to establish the optimal growth model.

After establishing the best-fitting growth curve model, a conditional LGC model was estimated, where the unconditional model was extended to include the effects of time-invariant variables (i.e., gender and school grade). The latent growth factors were regressed on the time-invariant variables. Finally, the model was extended to test the direct effects of a time-varying covariate (i.e., the COVID-19-related life disruption) on SESRL, above and beyond the influence of each individual’s underlying trajectory. In this case, each repeated measure of SESRL was regressed on the respective time-specific covariate.

As a sensitivity analysis, all growth models were re-estimated, including only participants with complete data across the three waves. The results were consistent with those obtained using FIML estimation, suggesting that the main findings were not dependent on the method used to handle missing data (see Supplementary Materials).

### 3.5. Results

#### 3.5.1. Longitudinal Measurement Invariance

The results of the longitudinal measurement invariance for SESRL and COVID-19-related life disruption are reported in Table S4. The configural model for both SESRL and COVID-19-related life disruption assessed over time provided adequate model fit, thus indicating that the same items measured the same hypothesized constructs across time points. Full metric and partial scalar invariance were established for both SESRL and COVID-19-related life disruption. For SESRL, partial scalar invariance was achieved by freeing the intercept of the item assessing the ability to remember the contents of teachers’ lessons, which was lower at T1. For COVID-19-related disruption, partial scalar invariance was obtained by freeing the intercepts of five items: family economic status at T1 (higher than in the other waves), relationships with parents at T2 (lower than in the other waves), relationships with peers as well as with partners at T2 (both higher than in the other waves), and free time at T2 (higher than in the other waves). This pattern likely suggests that the meaning of disruption in these domains may have varied across different stages of the pandemic.

These levels of invariance were considered adequate for the specific use of each measure in the growth models performed. For SESRL, whose latent means were compared over time through the estimation of the growth trajectory, partial scalar invariance, with two out of three item intercepts held equal across waves, still supports meaningful comparisons of factor means over time (Jang et al., 2017), although it calls for caution in interpreting latent mean change. For COVID-19-related life disruption, which was included as a time-varying covariate associated with SESRL within each wave, no comparison of factor means across waves was involved; the interpretation of these concurrent, wave-specific associations rests on the full metric invariance of the measure, ensuring that the construct was assessed on the same metric at each stage of the pandemic.

#### 3.5.2. Correlations Among Study Variables

Preliminarily, we checked for the distribution of variables. No variables approached skewness > |3| or kurtosis > |10|, indicating that the data followed a normal distribution (Weston & Gore, 2006). Descriptive statistics and correlations between all the study’s variables are shown in Table S5. As can be observed, all the study’s variables were significantly associated with each other, both concurrently and across time. More in detail, SESRL and COVID-19-related life disruption were negatively associated, both concurrently and across time. With respect to the associations with the control variables, we found that older students reported higher levels of SESRL at T2.

#### 3.5.3. Unconditional Model of Self-Efficacy for Self-Regulated Learning

The model fit indices and the results of the comparisons between the alternative growth curve models are shown in Table 2. Chi-square difference tests indicated that the linear growth model provided a significantly better fit than the no-growth model, Δχ^2^(3) = 30.96, *p* < 0.001. In contrast, the quadratic model did not fit the data significantly better than the linear model, Δχ^2^(1) = 0.91, *p* = 0.34. Given the absence of a significant improvement in fit, the lower AIC and BIC values of the linear model, and the limited number of assessment waves, the linear model was retained as the most parsimonious representation of change over time.

**Figure 3:**
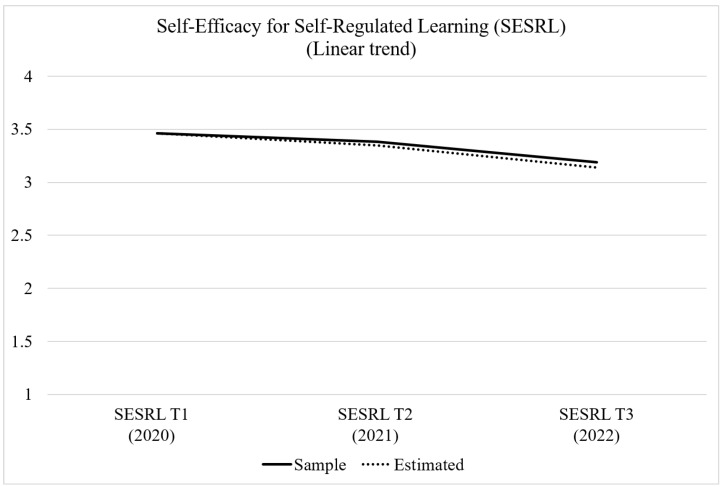
Sample and estimated growth trajectories for self-efficacy for self-regulated learning. Observed means were T1 = 3.46 [95% C.I.s: 3.37, 3.54], T2 = 3.38 [95% C.I.s: 3.28, 3.48], and T3 = 3.19 [95% C.I.s: 3.05, 3.33].

Both the fixed (M = 3.46, SE = 0.04, 95% C.I.s [3.38, 3.55], *p* < 0.001) and random (σ^2^ = 0.48, SE = 0.06, 95% C.I.s [0.36, 0.60], *p* < 0.001) effects of the intercept were statistically significant, suggesting significant interindividual differences in initial levels of SESRL. The mean of the linear slope was significant and negative (M = −0.14, SE = 0.03, 95% C.I.s [−0.21, −0.08], *p* < 0.001), indicating a linear decrease in SESRL over time (Figure 3). The variance in the linear slope was significant (σ^2^ = 0.08, SE = 0.04, 95% C.I.s [0.01, 0.15], *p* < 0.05), thus supporting interindividual variability in the rate of change (i.e., trajectories) in SESRL. The covariance between the intercept and linear slope was significant and negative (b = −0.12, SE = 0.04, 95% C.I.s [−0.20, −0.04], *p* < 0.01, r = −0.62), indicating that higher initial levels of SESRL were associated with a slower decline of SESRL over time.

#### 3.5.4. Conditional Growth Model: Effects of Time-Invariant and Time-Varying Covariates

As the unconditional LGC model showed significant interindividual variability in the intercept and slope, the linear growth model was extended by adding adolescents’ gender and school grade as time-invariant covariates. In addition, the concurrent value of the time-specific variable of COVID-19-related life disruption was evaluated by including it as a time-varying covariate in the same model (see Table 3).

**Figure 4:**
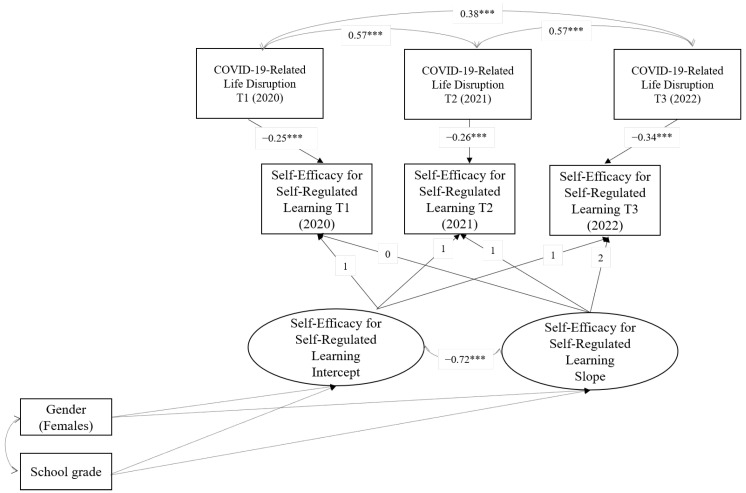
Effects of time-invariant and time-varying covariates on the growth curve of self-efficacy for self-regulated learning. The dashed lines are non-significant. Standardized estimates. *** *p* < 0.001.

Overall, the model showed marginal fit. As Figure 4 shows, neither gender (intercept: b = 0.05, SE = 0.09, 95% C.I.s [−0.12, 0.22]; slope: b = 0.01, SE = 0.07, 95% C.I.s [−0.13, 0.15]) nor school grade (intercept: b = 0.02, SE = 0.05, 95% C.I.s [−0.08, 0.12]; slope: b = 0.01, SE = 0.04, 95% C.I.s [−0.08, 0.09]) was significantly associated with the growth factors, whereas the effect of the time-varying covariate was significant and negative at each time point (T1: b = −0.39, SE = 0.07, 95% C.I.s [−0.53, −0.26]; T2: b = −0.36, SE = 0.05, 95% C.I.s [−0.47, −0.26]; T3: b = −0.45, SE = 0.09, 95% C.I.s [−0.64, −0.27]; all ps < 0.001), indicating that individuals with higher COVID-19-related life disruption at each stage of the pandemic reported significantly lower levels of SESRL.

### 3.6. Discussion

Grounded in Bandura’s self-efficacy theory, this study contributed to the literature on high-school students’ SESRL within the evolving context of the COVID-19 pandemic. Since Study 1 showed that students reported lower levels of SESRL during the first lockdown than when retrospectively referring to the pre-pandemic period, Study 2 examined the trajectory of SESRL beliefs across subsequent stages of the COVID-19 pandemic. Over two years, three time points corresponding to different stages of the pandemic, with different levels of restrictive measures in several life domains, were considered. Specifically, the study included data collected during the pandemic outbreak in Italy (T1) and examined the time-varying effect of COVID-19-related disruptions on SESRL growth trajectories at two additional time points, 12 and 24 months later (T2 and T3).

Results from the unconditional LGC models seem to primarily support the Cumulative Stress Model (Sameroff et al., 1998) hypothesis (1b) over the habituation or settlement hypothesis (1a)**,** revealing a significant linear decreasing trajectory of students’ SESRL during the different stages of the pandemic, albeit the modest model fit and the limited number of time points reduce the strength of the conclusions that can be drawn. A possible reason why increasing familiarity with online learning methods did not translate into a recovery of SESRL over time is that the conditions students faced kept changing across the three stages of the pandemic: The alternation between remote, blended, and in-person learning, together with persisting restrictions in several life domains, may have prevented students from consolidating stable learning routines on which to rebuild their confidence. Interestingly, the negative covariance between the intercept and the slope indicated that students with higher initial levels of SESRL experienced a slower decline in SESRL over time. This pattern is consistent with the buffering role attributed to self-efficacy beliefs within the social-cognitive framework (Bandura, 1997), suggesting that students who entered the pandemic with stronger confidence in their self-regulatory abilities may have been better equipped to preserve it as adversities accumulated.

To our knowledge, this is the first longitudinal study to investigate the pattern of SESRL beliefs across different stages of the pandemic in a sample of high-school students. The average decreasing trend in perceived SESRL as the pandemic evolved aligns with previous results (Adedoyin & Soykan, 2023) reporting lower academic self-efficacy during the pandemic and is consistent with Bandura’s view that self-efficacy beliefs are context-specific, do not represent a stable personality trait, and are responsive to environmental changes (Bergey et al., 2019). This may be particularly relevant in the unexpected context of emergency remote learning (Besser et al., 2022), where students were faced with novel tasks or experiences (Usher & Pajares, 2008). It is plausible to hypothesize that, given the suddenness of the COVID-19 pandemic, such a disruptive life event could have weakened the individual’s perception of mastery and control over their own life (Bandura, 1997), with significant implications for students’ SESRL beliefs. This could be especially understandable in the context of emergency online learning during the COVID-19 pandemic, as students had to rely on their personal beliefs as an internal strength to offset pandemic-related impacts and the concomitant learning loss (Wong & Yuen, 2023). However, given the absence of a pre-pandemic baseline as well as of a comparison group, the limited number of assessment waves across three widely spaced observations, and the substantial attrition over time which could be related to unobserved characteristics (e.g., lower school engagement, limited access to and supervision as well as support at home on remote learning; Alejo et al., 2024), this trend should be interpreted as a descriptive pattern of change within a pandemic-exposed cohort rather than as evidence of a pandemic-caused decline.

Moreover, lower levels of SESRL were still observed at the final wave of data collection, corresponding to the stage of the pandemic in which the health emergency was nearly overcome, and schools were returning to face-to-face learning activities. This pattern is compatible with a possible cascade process through which the pandemic could have triggered long-lasting effects on students’ SESRL beliefs. However, the data do not allow a direct test of the pandemic stress process, and alternative explanations related to developmental and educational changes over time should also be considered. Lower SESRL at T3 may reflect the return to the face-to-face learning model and its renewed evaluation standards, increasing academic demands and exam pressure, as well as age-related changes and general adolescent development. The inclusion of gender and school grade as time-invariant covariates, as well as COVID-19-related life disruption as a time-varying covariate, suggested that gender and school grade were associated with neither the initial levels nor the trajectories of SESRL (Hypothesis 2), whereas higher levels of COVID-19-related life disruption were associated with lower levels of students’ SESRL at each measurement point (Hypothesis 3). One possible interpretation of the non-significant effects of gender and school grade is that the pandemic acted as a pervasive stressful event. This result is somewhat different from Study 1, where students attending higher school grades reported larger differences between lockdown and retrospectively reported pre-pandemic SESRL. However, the two results are not directly comparable, as Study 1 focused on a retrospectively perceived change at the time of the first COVID-19 lockdown, whereas Study 2 modeled the average trajectory of SESRL across subsequent stages of the pandemic.

Furthermore, the finding that higher levels of disruption at a given stage of the pandemic were associated with lower SESRL at that same stage is in line with the cumulative stress perspective (Sameroff et al., 1998), suggesting that SESRL may be closely linked to adolescents’ perceptions of current challenges across different domains of daily life. However, it should be noted that these associations are concurrent and do not imply that pandemic-related disruption preceded changes in SESRL.

Overall, our findings could be understood in light of theoretical perspectives emphasizing the role of emotional states and moods on self-confidence judgments (Bandura, 1997). In line with Strasser et al. (2023), who suggested that negative emotional states are associated with the tendency to perceive situations as uncertain, uncontrollable, and threatening, adolescents experiencing higher levels of disruption across multiple life domains may have reported lower confidence in their ability to effectively manage learning activities. Similarly, Talsma et al. (2021) suggested that individuals may use their own feelings of arousal, uncertainty, anxiety, and stress associated with COVID-19-related changes as cues in judging their own efficacy (Usher & Pajares, 2006). Accordingly, some studies in the COVID-19 context reported an inversely proportional relationship between the perception of academic self-efficacy and negative emotions such as anxiety (Alemany-Arrebola et al., 2020; Navarro-Mateu et al., 2020). Such aspects, together with an increase in internalizing symptoms (i.e., anxiety/depression) and a decrease in adolescents’ regulatory self-efficacy for managing negative emotions due to the COVID stress (Skinner et al., 2023), should be considered when interpreting the possible long-lasting correlates of the COVID-19-pandemic on adolescents’ school adaptation. In general, it should be noted that the specific associations investigated in this study must be read in light of the marginal fit of the conditional model, which suggests caution in interpreting the covariate effects.

In addition, the presence of interindividual variability in the rate of SESRL change indicates that students’ trajectories differed significantly. Since genders and school grades did not account for this heterogeneity, other factors not considered in this study—such as prior academic achievement, socioeconomic status, digital access, family functioning, and mental health symptoms—may have made some students more vulnerable to the changing educational context. Future studies may benefit from implementing a person-centered approach based on students’ SESRL profiles—complementing the variable-centered approach used here—to identify subgroups of students with distinct SESRL trajectories and the individual and contextual factors associated with membership in the most unfavorable ones.

In summary, the findings of Study 2 show that students’ confidence in self-regulating their learning tended to decline, on average, as the pandemic evolved and covaried, at each stage, with the perceived disruption of their daily lives. Although descriptive in nature, this pattern suggests the sensitivity of SESRL beliefs to prolonged contextual adversity.

## 4. Conclusions

The studies provide insights into the existing literature on students’ academic self-efficacy in the context of emergency online learning by focusing on SESRL, which has been recognized as crucial for adapting to the novel learning environment (e.g., Biwer et al., 2021). Specifically, we combined a retrospective (Study 1) and a longitudinal (Study 2) research design. Together, these complementary approaches allowed us to examine both students’ reports of SESRL during the sudden shift to home-learning following the outbreak of the pandemic, compared with their retrospectively reported pre-pandemic SESRL, and the trajectories of SESRL as the pandemic evolved—a pattern of change that the previous literature, which mainly focused on the early stage of emergency online learning due to the pandemic (Talsma et al., 2021) and on undergraduate students (Aguilera-Hermida et al., 2021), had scarcely investigated in high-school students. In this regard, the Italian context—where restrictive measures were adopted early, and the school system was largely unprepared for the abrupt shift to remote learning (Vecchio et al., 2022)—represented a particularly informative setting for examining these processes. In most cases, online lessons were proposed in the form of experimental modules in a limited number of schools, rather than being established as a permanent solution to carry out learning activities. On the one hand, such a global paradigm shift toward online and remote learning enabled the continuity of learning processes while maintaining the social distancing restrictions; on the other hand, the digitalization of learning activities—which required moving from a physical classroom to a virtual, Internet-based environment—posed teachers and students with several difficulties strictly related to the use of digital platforms. Consistent with the context specificity of self-regulated learning and the unique characteristics of emergency remote learning (Biwer et al., 2021), we found that students reported lower levels of SESRL during the first lockdown (compared to retrospective pre-pandemic reports) and over time as the pandemic evolved. Furthermore, we explored individual and contextual factors potentially associated with SESRL during the pandemic, finding that perceived teacher support as well as temperamental self-regulatory abilities were associated with higher SESRL, suggesting that supportive teacher-student relationships and self-regulatory abilities may represent potentially relevant resources for maintaining confidence in managing learning-related activities during periods of educational disruption. In doing so, the present studies extend the limited literature on this topic, which has mainly examined academic self-efficacy as a predictor of achievement during the pandemic (Berman et al., 2022), by focusing instead on the individual and contextual factors associated with students’ SESRL.

Nevertheless, several strengths and limitations of the current studies need to be acknowledged. Among the main strengths, the longitudinal design allowed us to capture the average change in adolescents’ SESRL and to evaluate its concurrent association with COVID-19-related life disruption during three stages of the pandemic. However, it is important to note that our studies relied only on students’ retrospective perceptions of pre-pandemic SESRL, lacking pre-pandemic measures as well as a comparable cohort of students not impacted by the pandemic. These represent fundamental limitations that require caution when interpreting our findings. As suggested by previous studies (e.g., Blome & Augustin, 2015; Yamagata & Miura, 2023), retrospective methods for describing long-lasting and fluctuating events such as the pandemic could lead to a gap between an experience and its retrospection, which may have affected our results, as discussed in Study 1. For instance, consistent with previous evidence (e.g., Guazzini et al., 2022), while struggling with the COVID-19-related challenges in the educational context, students may have been more prone to remember their pre-pandemic SESRL as better than it actually was. Also, the absence of a comparison group prevents us from ascertaining whether the observed changes in SESRL across the different stages of the pandemic are specific to the COVID-19 pandemic or could reflect developmental aspects or the natural progression of time. Future studies, including pre-pandemic measures of SESRL as well as a comparable cohort of students evaluated before the pandemic outbreak, should verify whether the pattern of results reflects the specific challenges of the pandemic outbreak rather than developmental change, the mere passage of time, or retrospective bias.

Other methodological issues concern the operationalization of SESRL, which was limited to using only three items drawing on the original academic self-efficacy scale from Pastorelli et al. (2001). Consistent with the multidimensional nature of academic self-efficacy, the item selection was informed by previous studies (Pribeanu et al., 2023) and motivated by the aim to detect a specific domain of students’ self-efficacy, i.e., SESRL. However, we are aware of the potential psychometric risk for content validity due to an inaccurate selection of items, which could prevent meaningful coverage of the multidimensional SESRL construct. Moreover, relying on partial scalar invariance for a short scale limits the comparability of latent means across time and introduces some ambiguity in the interpretation of change. Although partial invariance can still be considered acceptable when a sufficient number of parameters are invariant (e.g., Byrne et al., 1989), this finding requires caution in interpreting the latent mean differences in SESRL across the different stages of the COVID-19 pandemic. This pattern may be partly explained by the specific contextual conditions characterizing each phase of the pandemic. In particular, variations in the level of restrictive measures across different life domains—especially in school routines—may have influenced how students interpreted SESRL items over time.

In addition, although COVID-19-related life disruption was conceptualized and analyzed as a global construct, this approach did not allow us to distinguish the unique contribution of specific domains of disruption (e.g., school, social, health, or economic domains) or to directly examine the extent to which the pandemic’s cumulative disruptive impact across multiple life contexts was associated with SESRL. Future studies should address these issues more explicitly.

As regards the longitudinal design we implemented to track students’ average change in SESRL over time, the use of only three measurement occasions provides limited evidence for developmental trajectories, as it restricts the reliable estimation of nonlinear growth patterns and does not allow for modeling individual variability in quadratic change. Specifically, as previous studies suggested (e.g., Cronin et al., 2026), relying on only three measurement points makes it difficult to determine whether any observed upward or downward shift in the SESRL trajectories was a random occurrence or reflected a meaningful peak/drop in the developmental process. In addition, as noted above, the fit of the conditional growth model was only marginal, suggesting that it may not fully capture the complexity of change over time. As such, the observed trajectories should be interpreted as indicative patterns rather than exact representations of developmental processes. One of the major methodological limitations of our study was the substantial attrition across the three assessment waves. As suggested by previous studies (e.g., Graca et al., 2023; T. Yu et al., 2022), online longitudinal surveys conducted during the pandemic, such as our study, were particularly vulnerable to participant attrition over time, potentially affecting the robustness of developmental estimates. In our study, the missingness was addressed using FIML, which has been demonstrated to be robust in longitudinal designs under the missing-at-random assumption (Tang & Tong, 2023), and a complete-case sensitivity analysis (see Supplementary Materials) yielded results consistent with the FIML estimates, indicating that the main findings were not dependent on the method used to handle missing data. However, neither approach can exclude a missing-not-at-random pattern in which dropout depends on unobserved values. Accordingly, the attrition rate substantially limits the strength of the longitudinal conclusions we can draw. Future research during stressful events might benefit from employing statistical methods, including stratification-based techniques, multiple imputations, and inverse probability-of-censoring weighted estimation, to mitigate the effects of attrition bias (Howe et al., 2016).

In addition, all measurements in the two studies relied exclusively on adolescent self-reporting. In general, the use of a unique source of information requires caution in interpreting results, as they could be altered due to potentially shared (or common) method variance. Specifically, shared method bias related to the use of self-report tools may produce biased estimates of the reliability and validity of the examined constructs and erroneous parametric estimates of the relationships between two constructs (Kaltsonoudi et al., 2022). Future studies may benefit from utilizing a multi-informant approach, e.g., by involving teachers in the measurement of support or parents in the assessment of temperamental constructs.

Another limitation concerns the generalizability of the results, as all subjects in the present studies came from the same geographical area in Southern Italy, and the sample is gender-imbalanced. Although most studies rely on samples from specific geographical areas, we are aware that the COVID-19 pandemic may have differentially impacted other subgroups within the Italian population, as well as other countries around the world, due to the different spread of infection and death rates, as well as different restriction rules in various life domains. Further, although we included adolescents’ gender as a control variable in all the main analyses, it should be noted that previous studies have found that females generally reported higher levels of academic self-efficacy (e.g., C. Huang, 2013) and were most affected by the pandemic crisis (Giudice et al., 2022). Future studies involving different samples from diverse cultural contexts and implementing a gender-balanced sampling strategy would help verify the generalizability of our results.

Finally, as discussed in Study 2, it would be useful for future studies to take into account the role of other relevant individual, family, and educational variables, including prior academic achievement, socioeconomic status, digital access, peer support, family functioning, and mental health symptoms, which may be associated with both COVID-19-related life disruption and SESRL and could contribute to adolescents’ school adjustment during periods of disruption (e.g., Paizan et al., 2022; Strasser et al., 2023).

Overall, given that the pandemic crisis may be considered globally as a salient example of educational and societal disruption and that online learning, from being an emergency response, has evolved into a widespread educational practice shaping post-COVID blended learning environments (Zhang & Gillespie, 2023), our study’s findings could provide useful insights for school stakeholders. In terms of practical implications, although the present studies did not evaluate specific interventions, the observed associations suggest that students’ self-regulatory abilities and teacher support may represent promising targets for school-based programs aimed at supporting students’ confidence in their academic capabilities (Bandura, 1997), especially when exposed to abrupt changes in their lives, as those experienced during the pandemic. More specifically, the teacher support measure assessed both academic support (e.g., encouragement and assistance with schoolwork) and personal support (e.g., teachers’ availability and care toward students), whereas effortful control captured attentional regulation, persistence, and inhibitory control. Educational approaches building on these resources, along with autonomous and student-centered learning, may help students to take control of their own learning experience, become motivated to learn, engage with the lesson and adapt more effectively to changing educational contexts, including online learning environments (Mohtar & Md Yunus, 2022).

## Figures and Tables

**Table 1:** Main and interaction effects on self-efficacy for self-regulated learning (SESRL) during the first COVID-19 pandemic lockdown.

**Main Effects of Predictors on SESRL During the First COVID-19 Pandemic Lockdown**						
	**Model 1**		**Model 2**		**Model 3**	
	**B [95% C.I.s]**	**R^2^**	**B [95% C.I.s]**	**R^2^**	**B [95% C.I.s]**	**R^2^**
Retrospectively reported pre-pandemic SESRL	0.63 *** [0.57, 0.70]	0.31 ***	0.64 *** [0.58, 0.71]	0.33 ***	0.51 *** [0.44, 0.58]	0.41 ***
Gender (male vs. female)			0.02 [−0.10, 0.14]		0.04 [−0.07, 0.15]	
School grade (9th–13th grade)			−0.09 *** [−0.13, −0.05]		−0.07 *** [−0.11, −0.03]	
COVID-19-related life disruption					−0.34 *** [−0.43, −0.24]	
Teacher support					0.14 *** [0.06, 0.22]	
Effortful control					0.26 *** [0.13, 0.40]	
F	358.63 ***		129.80 ***		92.09 ***	
ΔR^2^	0.31 ***		0.02 ***		0.08 ***	
**Interaction Effects of Predictors on SESRL During the First COVID-19 Pandemic Lockdown**						
	**Model 4**					
	**B [95% C.I.s]**			**ΔR^2^**		
Retrospectively reported pre-pandemic SESRL × gender	0.01 [−0.12, 0.14]			0.00		
F	0.01					
	**Model 5**					
Retrospectively reported pre-pandemic SESRL × school grade	−0.00 [−0.05, 0.04]			0.00		
F	0.01					
	**Model 6**					
Retrospectively reported pre-pandemic SESRL × COVID-19-related life disruption	−0.19 *** [−0.29, −0.09]			0.01 ***		
F	12.97 ***					
	**Model 7**					
Retrospectively reported pre-pandemic SESRL × teacher support	0.09 * [0.01, 0.17]			0.00 *		
F	4.73 *					
	**Model 8**					
Retrospectively reported pre-pandemic SESRL × effortful control	0.04 [−0.08, 0.16]			0.00		
F	0.40					

Notes. x = interaction with; * *p* < 0.05, *** *p* < 0.001.

**Table 2:** Model fit for unconditional growth curve models and comparisons between alternative models.

	No Growth					Linear					Quadratic				
	χ^2^ (df)	CFI	RMSEA	BIC	AIC	χ^2^ (df)	CFI	RMSEA	BIC	AIC	χ^2^ (df)	CFI	RMSEA	BIC	AIC
Self-efficacy for self-regulated learning	31.91 (6)	0.71	0.10	2104.270	2091.909	0.95 (3)	1.00	0.00	2091.668	2066.947	0.04 (2)	1.00	0.00	2096.877	2068.035

Notes. Goodness of fit indices of the best-fitting model are in bold. df = degrees of freedom. The variance of the quadratic slope was fixed to zero, meaning that the model reflects only the average nonlinear trend, without considering individual differences. The quadratic effect should therefore be interpreted with caution.

**Table 3:** Fit indices of the conditional growth curve model with time-invariant and time-varying covariates.

	Linear					
	χ^2^ (df)	*p*	CFI	RMSEA	BIC	AIC
Self-efficacy for self-regulated learning	40.73 (11)	<0.001	0.90	0.08	5187.98	5052.01

Notes. df = degrees of freedom. Following standard latent growth curve modeling procedures, covariances between each repeated measure of SESRL and the time-invariant covariates, as well as between the intercept and slope and the time-varying covariate, were fixed to zero.

## Data Availability

De-identified data are available from the corresponding author upon reasonable request, subject to ethical approval and compliance with data protection regulations.

## Supplementary Materials

The following supporting information can be downloaded at https://www.mdpi.com/article/10.3390/bs16071242/s1. Table S1: Items, descriptive statistics, and standardized factor loadings for self-efficacy for self-regulated learning (SESRL). Table S2: Items, descriptive statistics, and standardized factor loadings for the COVID-19-related life disruption scale. Table S3: Correlations, means (M), and standard deviations (SDs). Table S4: Measurement invariance tests across time points. Table S5: Correlations, means (M) and standard deviations (SDs). Table S6: Model fit for unconditional growth curve models and comparisons between alternative models. Table S7: Fit indices of conditional growth curve model with time-invariant and time-varying covariates. Figure S1: Missingness analysis—density plots across groups. Figure S2: Sample and estimated growth trajectories for self-efficacy for self-regulated learning. Figure S3: Effects of time-invariant and time-varying covariates on the growth curve of self-efficacy for self-regulated learning.
