# Supplementary material for: Self-Efficacy for Self-Regulated Learning Across Different Stages of the COVID-19 Pandemic: A Three-Wave Study with High-School Students
Source: Behav Sci (Basel). 2026 Jul 21;16(7):1242. doi: 10.3390/bs16071242 (PMC13403404; doi:10.3390/bs16071242)
Supplement: Supplementary file 1 [file behavsci-16-01242-s001.zip › Figure S2_rev01.pdf]

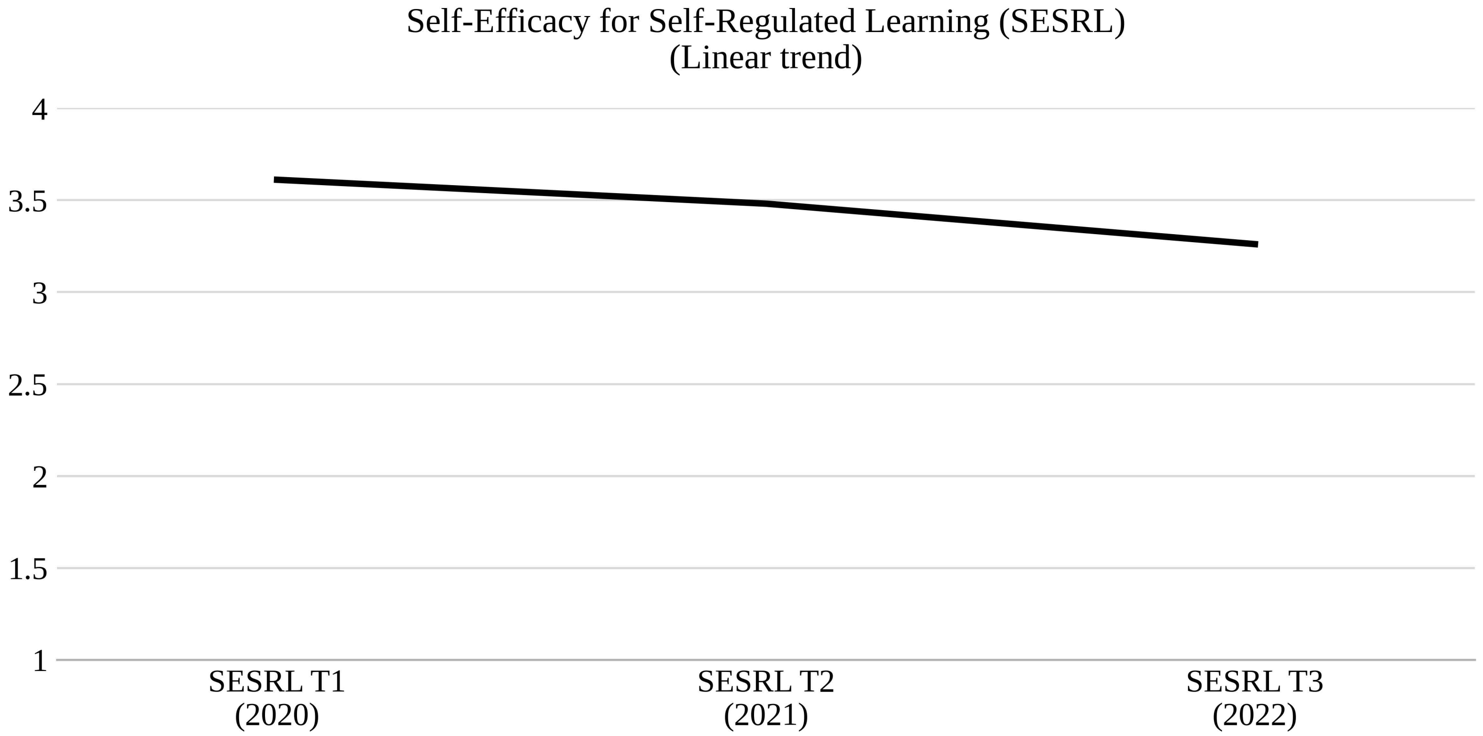

Figure s2. Sample and estimated growth trajectories for self-efficacy for self-regulated learning. Findings obtained by applying the listwise deletion method (N = 102).
