# Supplementary material for: Self-Efficacy for Self-Regulated Learning Across Different Stages of the COVID-19 Pandemic: A Three-Wave Study with High-School Students
Source: Behav Sci (Basel). 2026 Jul 21;16(7):1242. doi: 10.3390/bs16071242 (PMC13403404; doi:10.3390/bs16071242)
Supplement: Supplementary file 1 [file behavsci-16-01242-s001.zip › Tables S1-S7_rev.pdf]

Table S1. Items, descriptive statistics, and standardized factor loadings for self-efficacy for self-regulated learning (SESRL)

**Item**	**Time**	**M**	**SD**	**λ**
How well can you concentrate when studying?	Pre-pandemic (retrospective)	3.32	1.04	0.74
How well can you organize your schoolwork?	Pre-pandemic (retrospective)	3.75	0.98	0.82
How well can you remember the contents of teachers' lessons?	Pre-pandemic (retrospective)	3.78	0.99	0.69
How well can you concentrate when studying?	Lockdown	3.23	1.06	0.86
How well can you organize your schoolwork?	Lockdown	3.51	1.07	0.88
How well can you remember the contents of teachers' lessons?	Lockdown	3.41	1.11	0.74

Note. N = 802. λ = standardized factor loading. All factor loadings were statistically significant ($p < 0.001$).

Table S2. Items, descriptive statistics, and standardized factor loadings for the COVID-19-related life disruption scale

**Item**	**M**	**SD**	**λ**
Relationships with parents	1.58	0.88	.54
Relationships with peers	2.01	0.98	.52
Romantic relationships	1.87	1.09	.37
School activities	2.61	1.02	.43
Free time	2.81	1.12	.34
Physical health	2.19	1.02	.59
Psychological health	2.47	1.07	.65
Academic performance	1.80	0.89	.52
Family economic status	2.06	0.97	.37

Note. $N = 802$. λ = Standardized factor loading. All factor loadings were statistically significant ($p < 0.001$).

Table S3. Correlations, means (M) and standard deviations (SDs)

**Measures**	**1**	**2**	**3**	**4**	**5**	**6**	**7**
1. Gender (male *vs*. female)	1						
2. School grade (9th *vs*. 13th grade)	0.01	1					
3. Pre-pandemic SESRL (retrospective reports)	0.09*	0.05	1				
4. SESRL during the first COVID-19 pandemic lockdown	0.06	-0.11**	0.56***	1			
5. COVID-19-related life disruption	0.01	0.07	-0.11**	-0.31***	1		
6. Teacher support	0.07	-0.06	0.24***	0.28***	-0.18***	1	
7. Effortful control	-0.02	-0.06	0.50***	0.45***	-0.31***	0.24***	1
M (*SD*)	-	-	3.61 (0.84)	3.38 (0.96)	2.15 (0.57)	3.80 (0.70)	3.40 (0.47)

Notes. SESRL = Self-efficacy for self-regulated learning. *$p < 0.05$, **$p < 0.01$, ***$p < 0.001$.

Table S4. Measurement invariance tests across time-points

	χ^2 (df)	$\Delta SB\chi^2$ (Δdf)	$p(\Delta SB\chi^2)$	CFI	RMSEA
Self-efficacy for self-regulated learning					
Configural model	17.878 (15)			0.99	0.02
Metric model	24.503 (19)	6.62 (4)	0.16	0.99	0.03
Scalar model	58.947 (23)	34.44 (4)	< 0.001	0.96	0.06
Partial scalar model	31.325 (22)	6.82 (3)	0.08	0.99	0.03
COVID-19-related life disruption					
Configural model	465.323 (293)			0.90	0.04
Metric model	484.115 (309)	18.79 (16)	0.28	0.90	0.04
Scalar model	556.292 (325)	72.18 (16)	< 0.001	0.87	0.04
Partial scalar model	502.260 (320)	18.15 (11)	0.08	0.90	0.04

Notes. df = degrees of freedom; $\Delta SB\chi^2$ = difference in Satorra–Bentler chi-square statistic between nested models; Δdf = difference in degrees of freedom between nested models; $p(\Delta SB\chi^2)$ = p-value of the $\Delta SB\chi^2$.

Table S5. Correlations, means (M) and standard deviations (SDs)

Measures	1	2	3	4	5	6	7	8
1. Gender (male *vs*. female)	1							
2. School grade (9th *vs*. 13th grade)	0.07	1						
3. SESRL T1	0.01	-0.01	1					
4. SESRL T2	0.10	0.16**	0.46***	1				
5. SESRL T3	-0.06	-0.12	0.35***	0.43***	1			
6. COVID-19-related life disruption T1	0.02	-0.00	-0.27***	-0.15*	-0.41***	1		
7. COVID-19-related life disruption T2	0.09	-0.08	-0.25***	-0.29***	-0.38***	0.55***	1	
8. COVID-19-related life disruption T3	0.11	0.05	-0.17*	-0.34***	-0.41***	0.37***	0.57***	1
M (*SDs*)	-	-	3.46 (0.91)	3.38 (0.80)	3.19 (0.82)	2.13 (0.57)	2.42 (0.56)	2.33 (0.60)

Notes. SESRL = Self-efficacy for self-regulated learning. *$p < 0.05$, **$p < 0.01$, ***$p < 0.001$.

Table S6. Model fit for unconditional growth curve models and comparisons between alternative models

	No growth					Linear					Quadratic				
	χ^2 (df)	CFI	RMSEA	BIC	AIC	χ^2 (df)	CFI	RMSEA	BIC	AIC	χ^2 (df)	CFI	RMSEA	BIC	AIC
Self-efficacy for self-regulated learning	22.13 (6)	0.70	0.16	750.148	742.273	**0.72 (3)**	**1.00**	**0.00**	**742.612**	**726.862**	0.35 (2)	1.00	0.00	746.868	728.494

Notes. Findings obtained by applying the listwise deletion methos (N = 102). Goodness of fit indices of the best-fitting model are in bold. df = degrees of freedom. *The variance of the quadratic slope was fixed to zero, meaning that the model reflects only the average nonlinear trend, without considering individual differences. The quadratic effect should therefore be interpreted with caution.

Table S7. Fit indices of conditional growth curve model with time-invariant and time-varying covariates

Notes. Findings obtained by applying the listwise deletion methos (N = 102). df = degrees of freedom. *Covariances between each repeated measure of SESRL and the time-

	**Linear**					
	**$\chi 2$ (df)**	**p**	**CFI**	**RMSEA**	**BIC**	**AIC**
Self-efficacy for self-regulated learning	32.32 (11)	< 0.001	0.88	0.14	1648.998	1562.374

invariant covariates as well as between the intercept and slope and the time-varying covariate were fixed to zero.
