# Supplementary figures and images for: Self-Efficacy for Self-Regulated Learning Across Different Stages of the COVID-19 Pandemic: A Three-Wave Study with High-School Students
Source: Behav Sci (Basel). 2026 Jul 21;16(7):1242. doi: 10.3390/bs16071242 (PMC13403404; doi:10.3390/bs16071242)

[A]

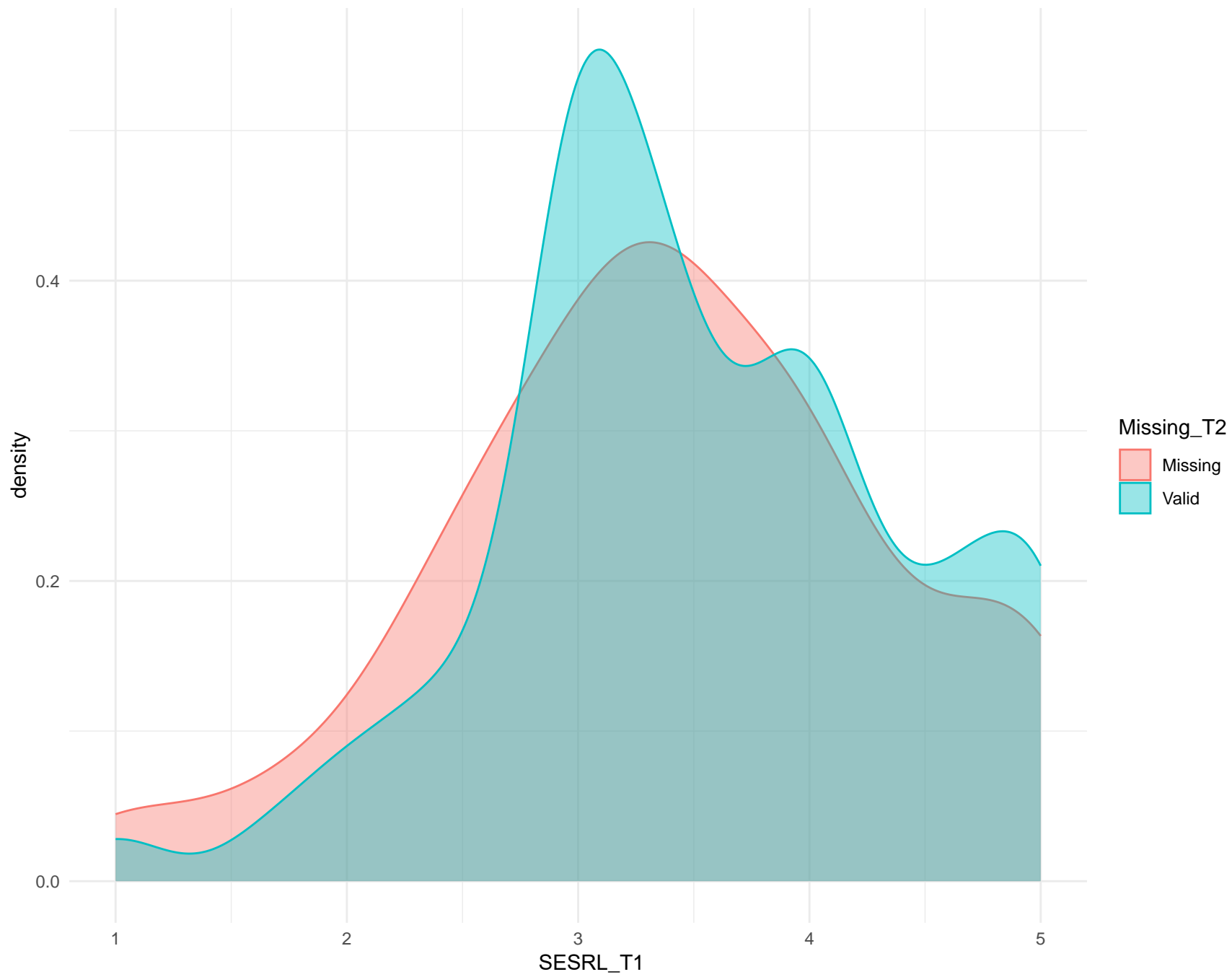

[B]

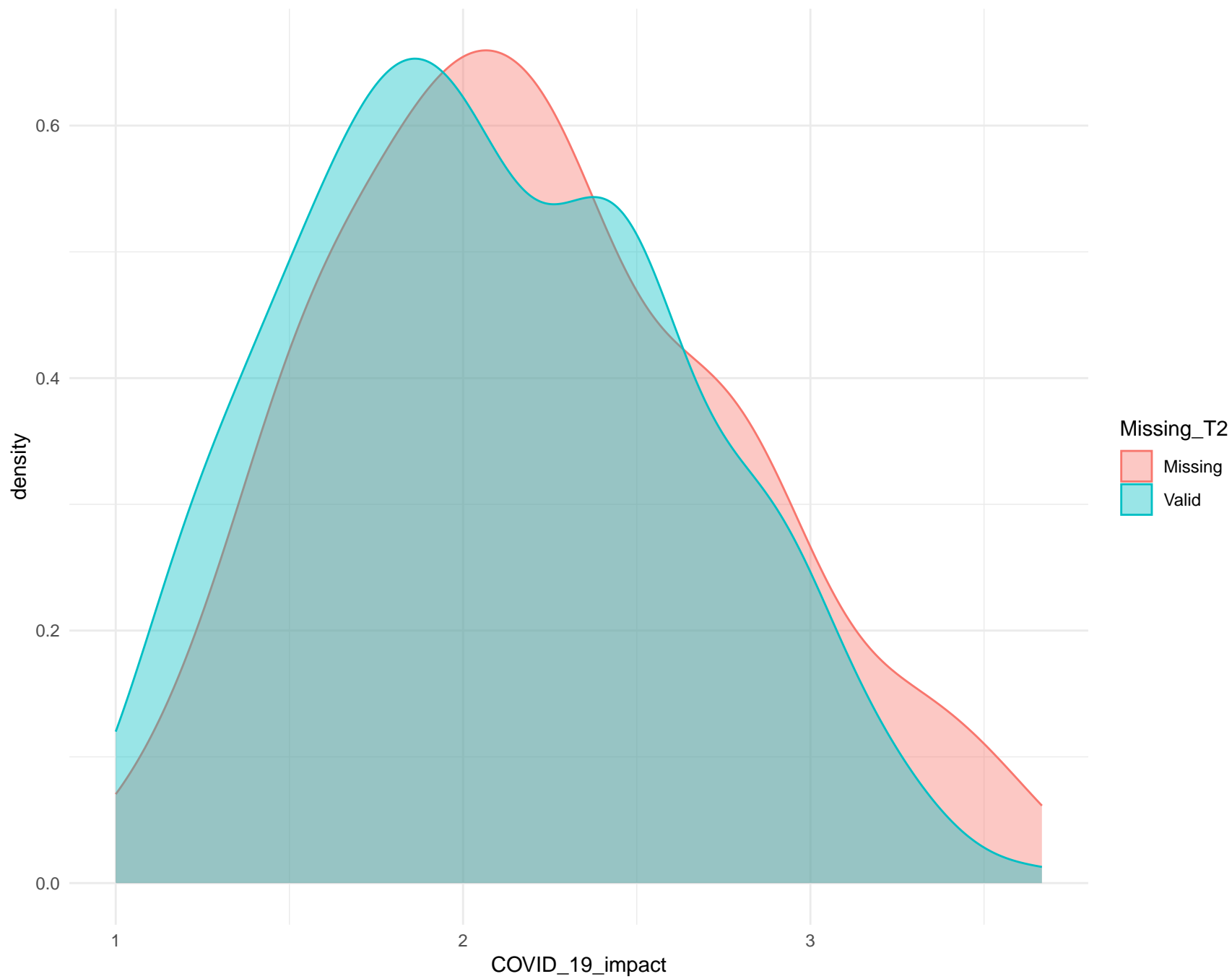

[C]

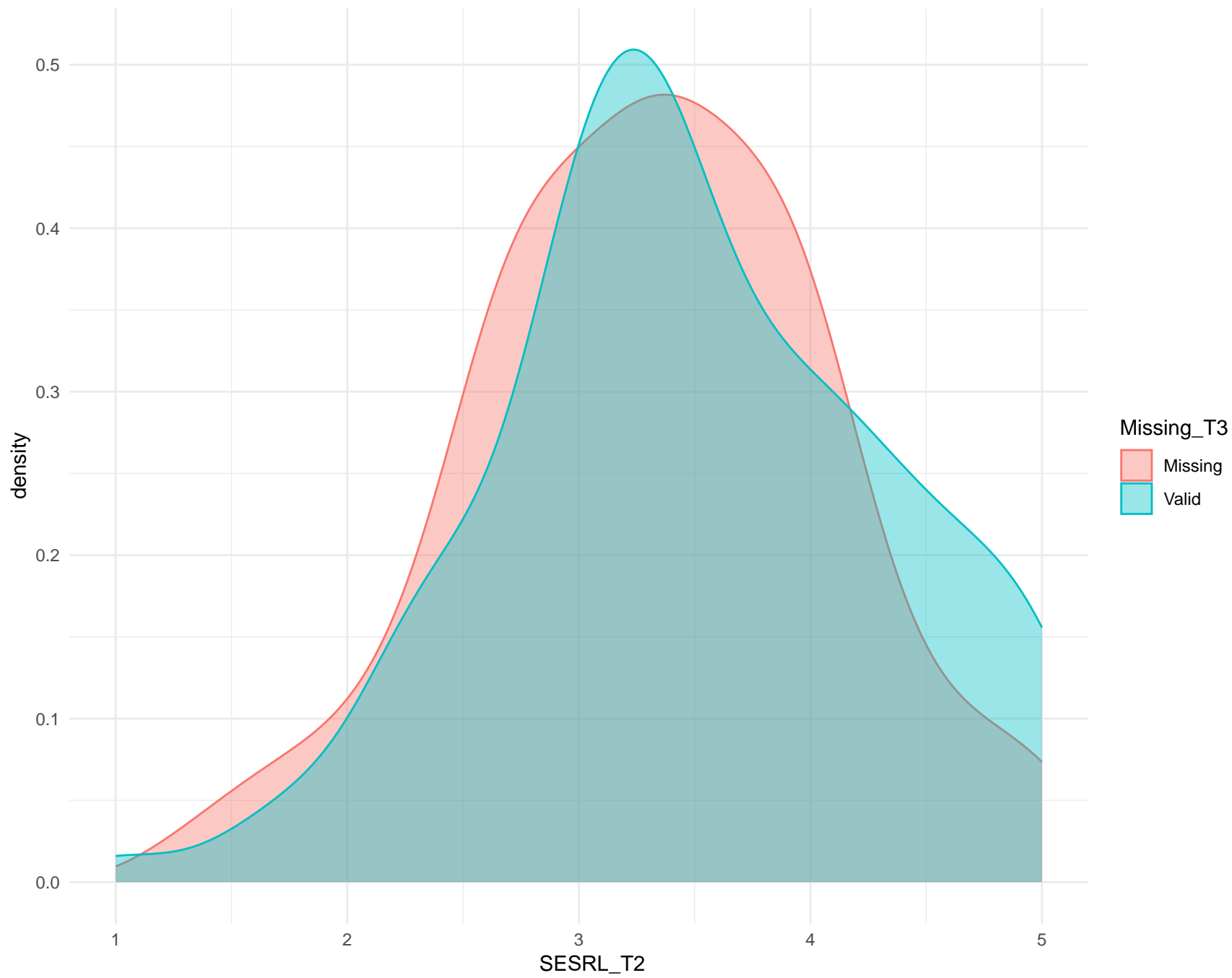

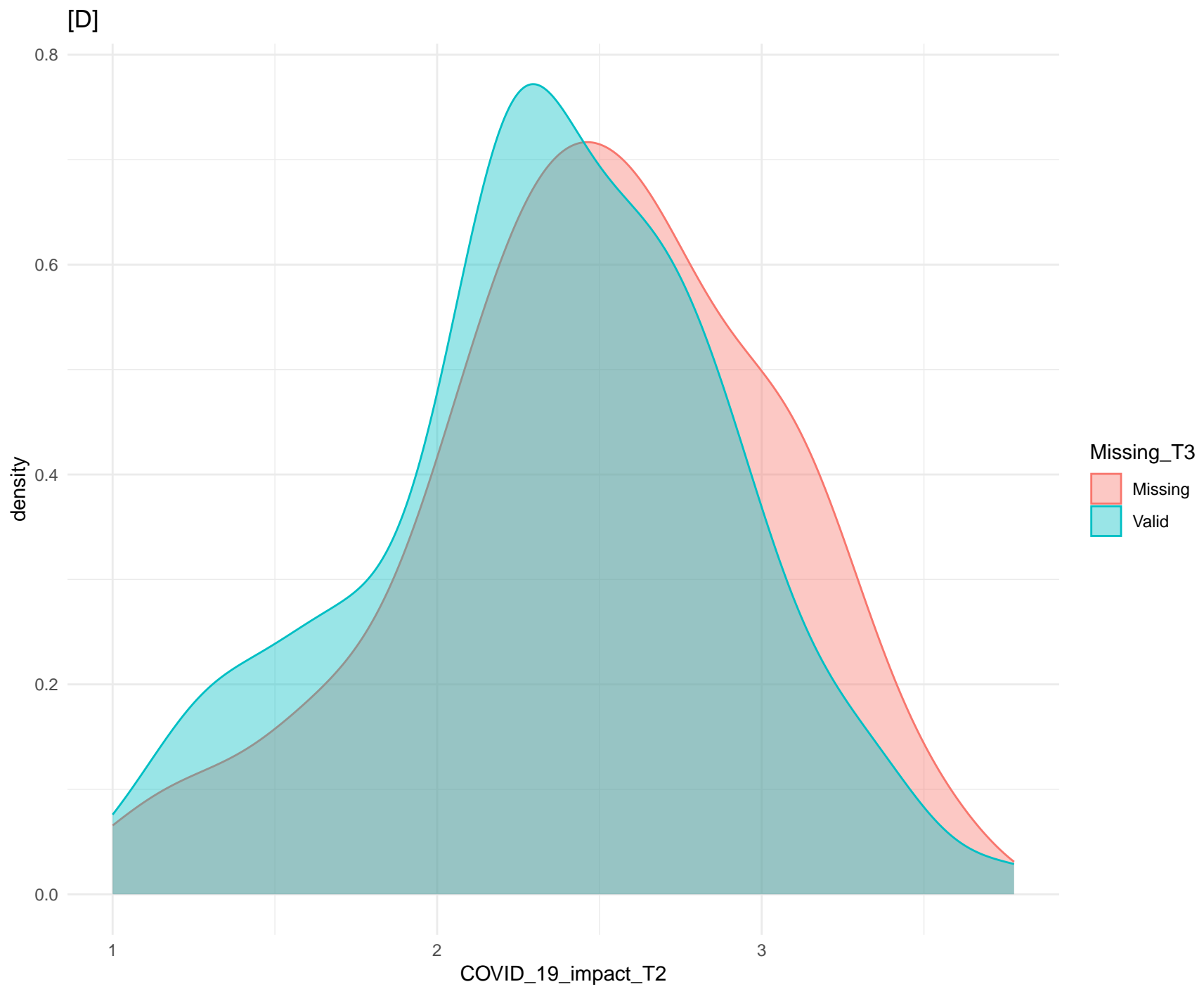

[E]

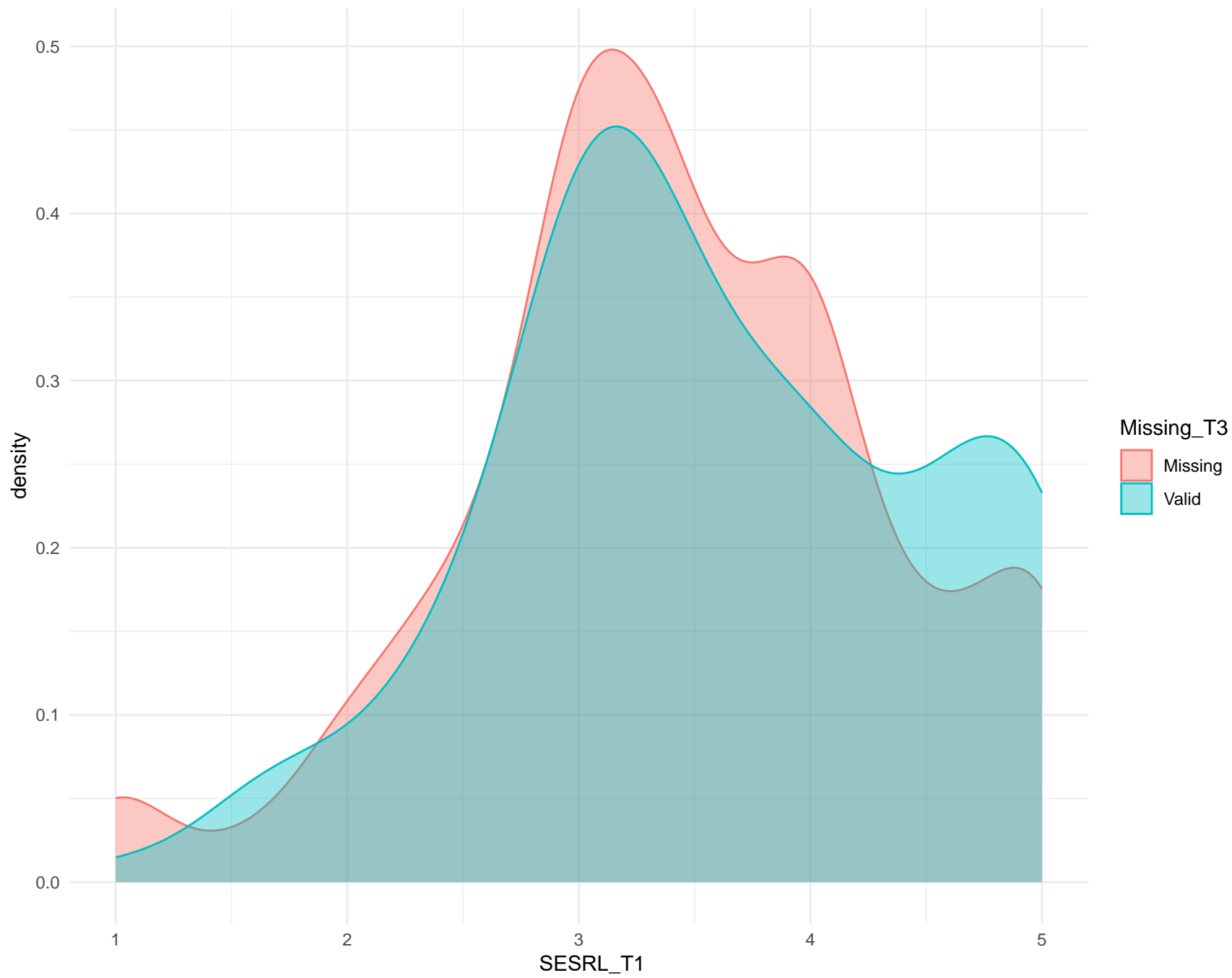

[F]

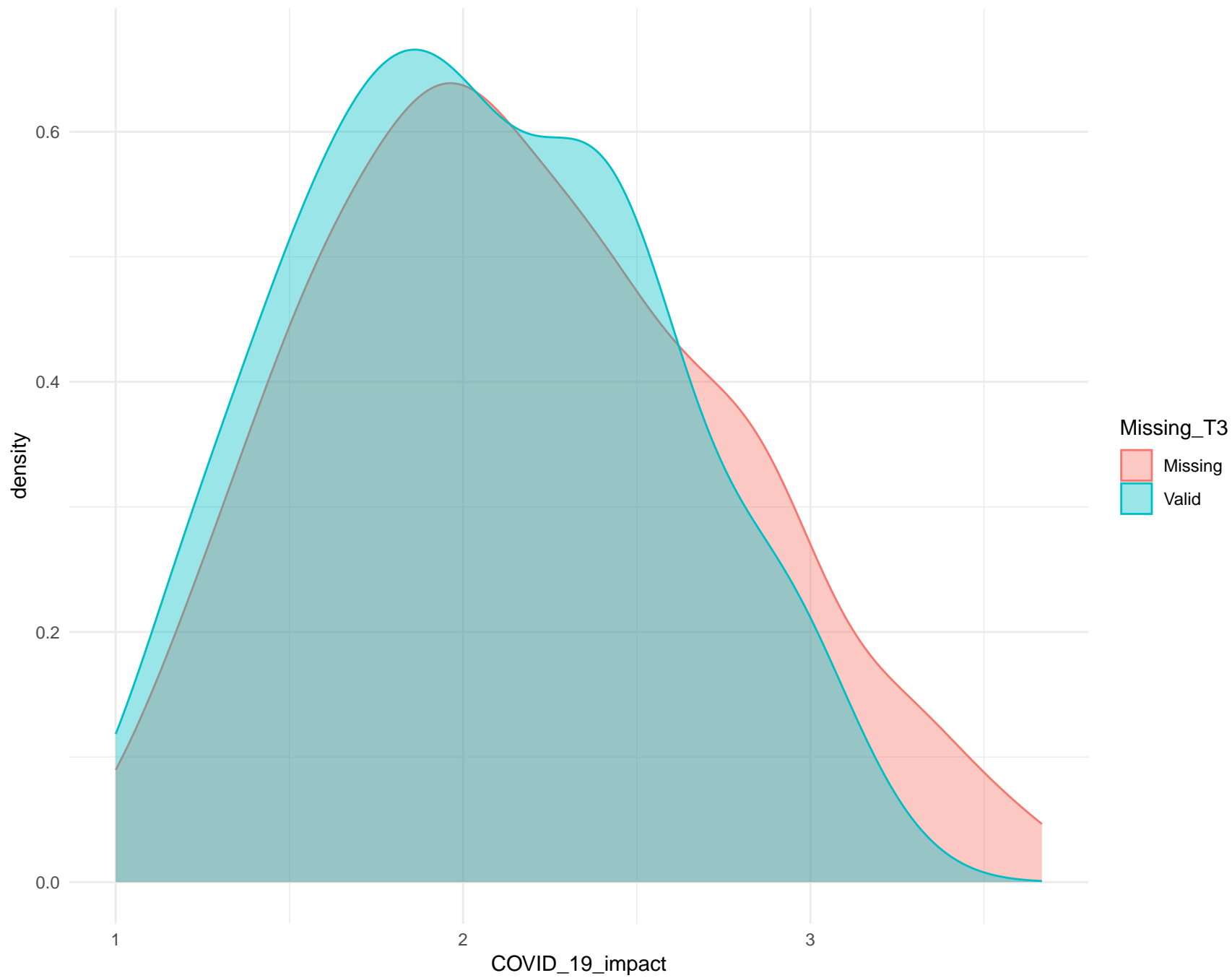

[G]

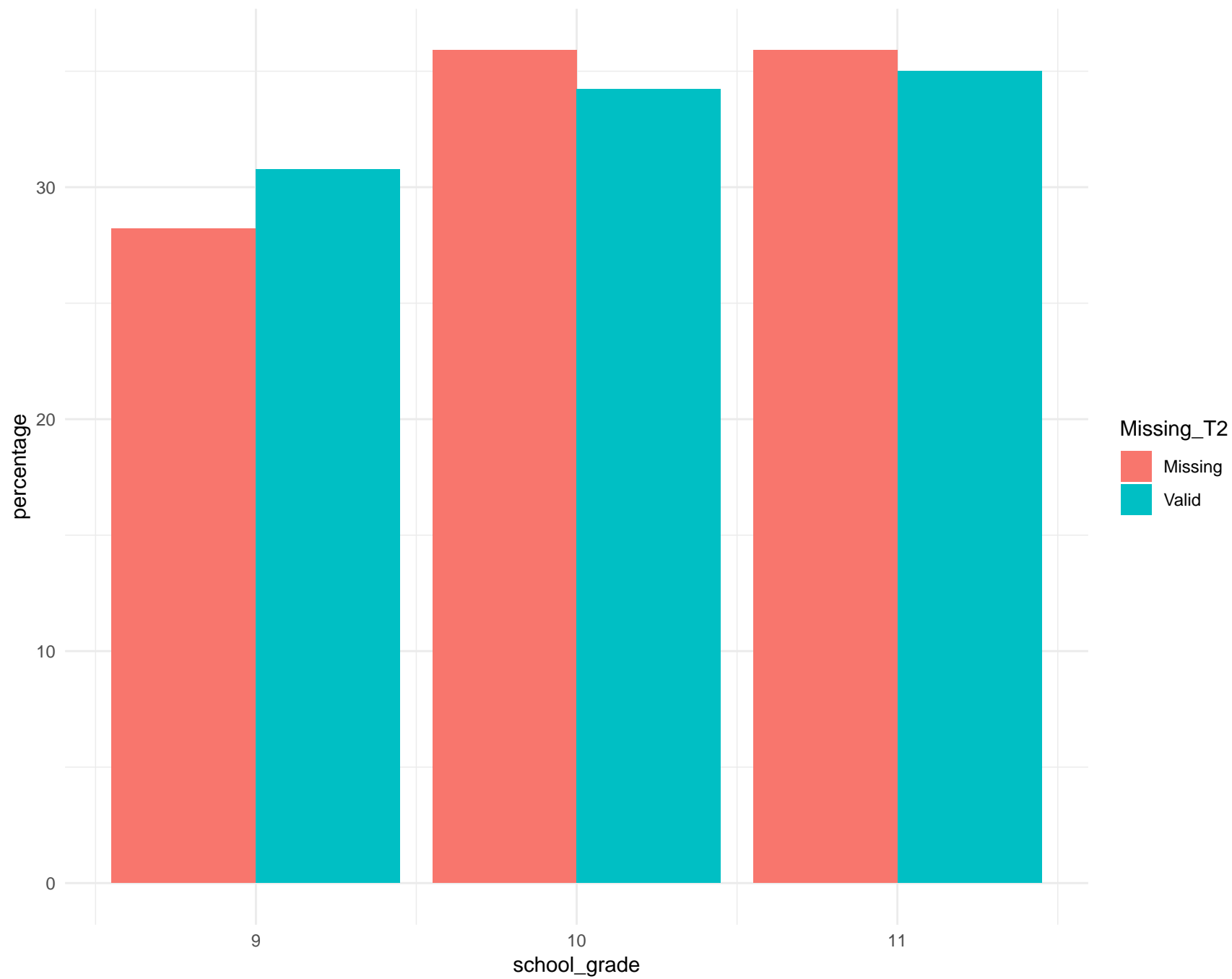

[H]

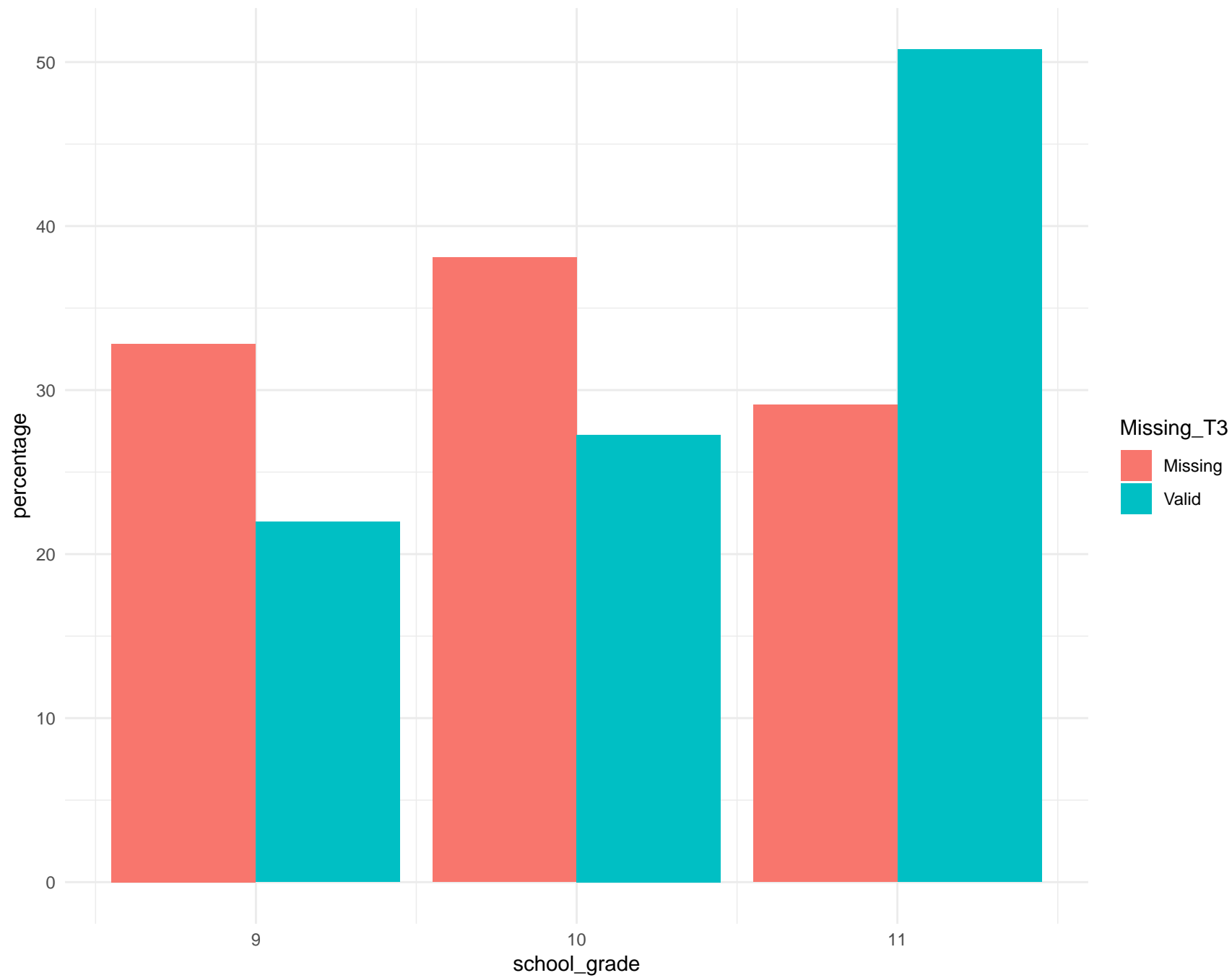

[1]

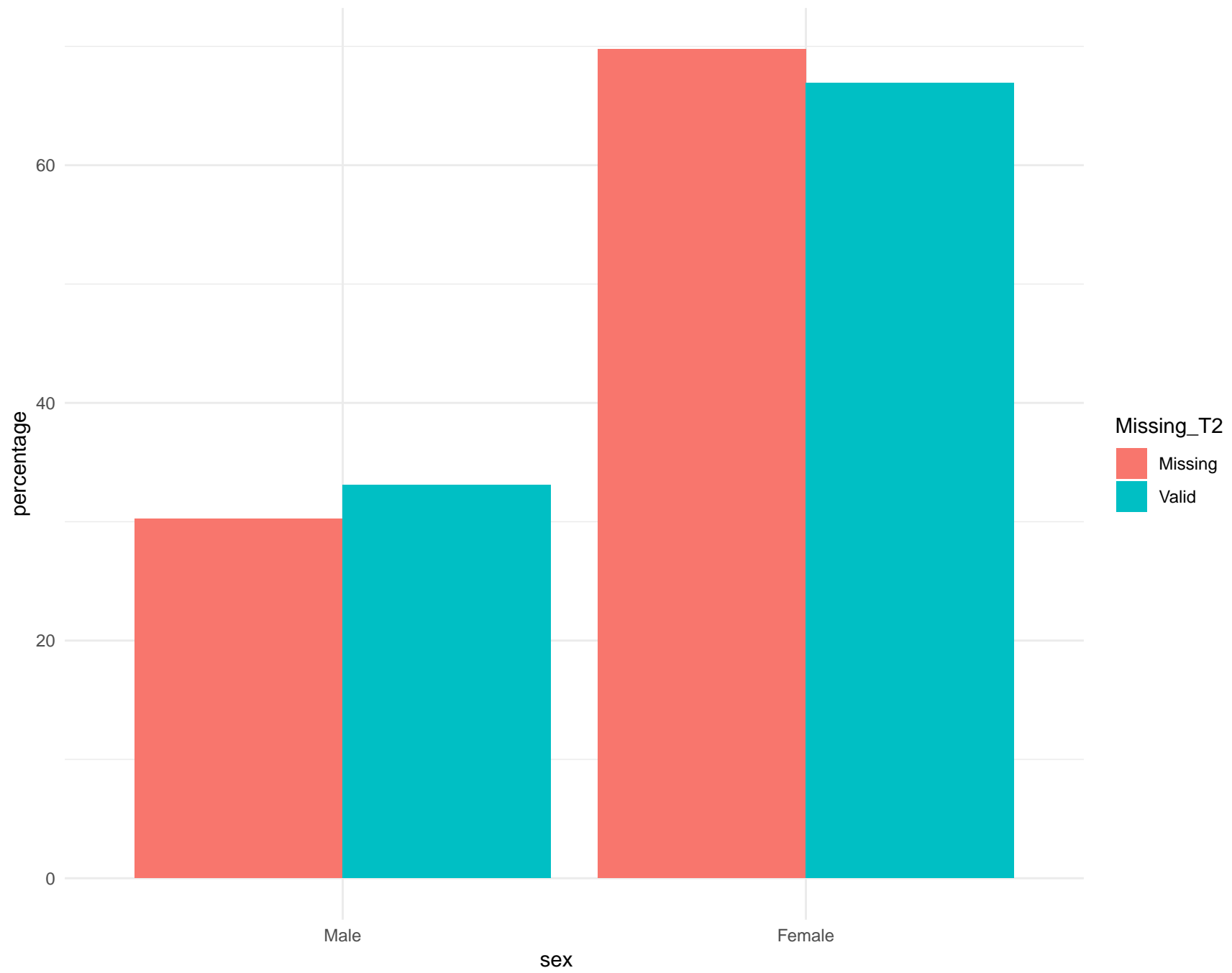

[J]

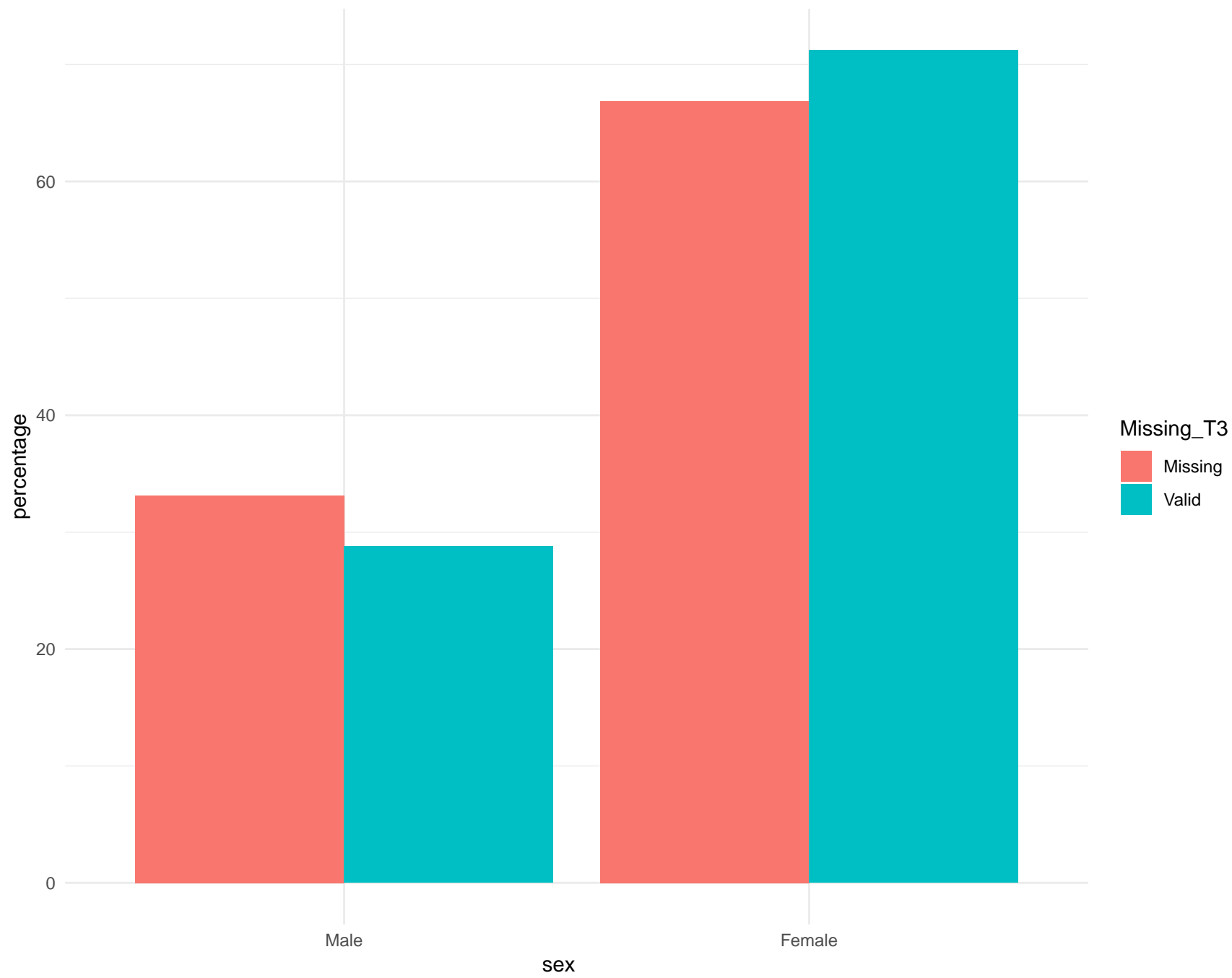

Supplement: Supplementary file 1 [file behavsci-16-01242-s001.zip › Figure S1_rev.pdf]
